# Nanofiltration Membranes for Efficient Lithium Extraction from Salt-Lake Brine: A Critical Review

**DOI:** 10.1021/acsenvironau.4c00061

**Published:** 2024-11-20

**Authors:** Ming Yong, Yang Yang, Liangliang Sun, Meng Tang, Zhuyuan Wang, Chao Xing, Jingwei Hou, Min Zheng, Ting Fong May Chui, Zhikao Li, Zhe Yang

**Affiliations:** † Dow Centre for Sustainable Engineering Innovation, School of Chemical Engineering, The University of Queensland, Brisbane, QLD 4072, Australia; ‡ Department of Chemical and Biological Engineering, Monash University, Clayton, VIC 3800, Australia; § Suzhou Industrial Park Monash Research Institute of Science and Technology, Suzhou, 215000, Jiangsu Province, China; ∥ Department of Civil Engineering, The University of Hong Kong, Pokfulam, Hong Kong 999077, SAR China; ⊥ School of Chemical Engineering, The University of Queensland, St Lucia, QLD 4072, Australia; # Water Research Centre, School of Civil and Environmental Engineering, University of New South Wales, Sydney, New South Wales, 2052, Australia

**Keywords:** nanofiltration, lithium extraction, membrane modification, process optimization, machine learning, system-scale analysis, lithium recovery, lithium purity

## Abstract

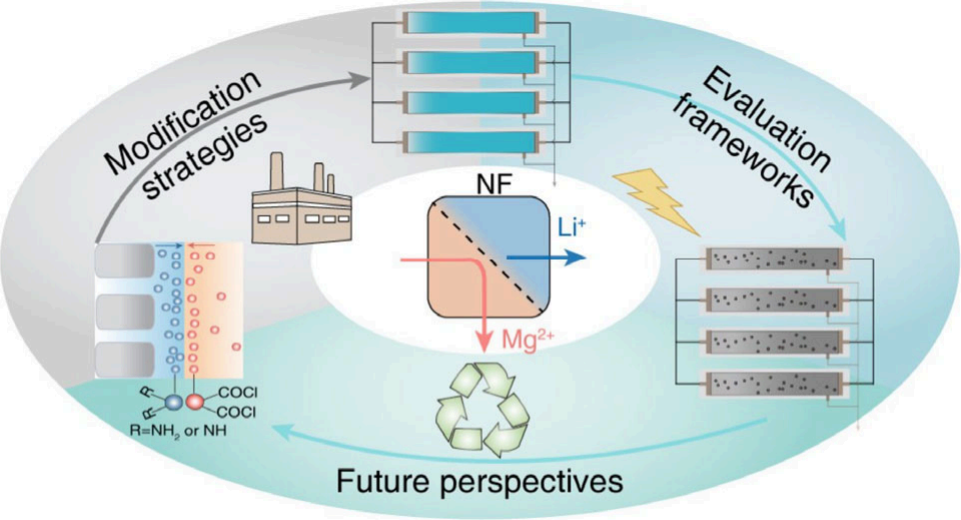

The global transition to clean energy technologies has escalated the demand for lithium (Li), a critical component in rechargeable Li-ion batteries, highlighting the urgent need for efficient and sustainable Li^+^ extraction methods. Nanofiltration (NF)-based separations have emerged as a promising solution, offering selective separation capabilities that could advance resource extraction and recovery. However, an NF-based lithium extraction process differs significantly from conventional water treatment, necessitating a paradigm shift in membrane materials design, performance evaluation metrics, and process optimization. In this review, we first explore the state-of-the-art strategies for NF membrane modifications. Machine learning was employed to identify key parameters influencing Li^+^ extraction efficiency, enabling the rational design of high-performance membranes. We then delve into the evolution of performance evaluation metrics, transitioning from the traditional permeance-selectivity trade-off to a more relevant focus on Li^+^ purity and recovery balance. A system-scale analysis considering specific energy consumption, flux distribution uniformity, and system-scale Li^+^ recovery and purity is presented. The review also examines process integration and synergistic combinations of NF with emerging technologies, such as capacitive deionization. Techno-economic and lifecycle assessments are also discussed to provide insights into the economic viability and environmental sustainability of NF-based Li^+^ extraction. Finally, we highlight future research directions to bridge the gap between fundamental research and practical applications, aiming to accelerate the development of sustainable and cost-effective Li^+^ extraction methods.

## 1 Introduction

The surging demand for critical metals, driven by the rapid growth of industrial sectors such as energy storage, advanced manufacturing, and emerging clean energy technologies, is straining the supply of their finite mineral reserves.^1−3^ This escalating demand, coupled with anticipated decreases in supplies and declining ore grades, raises concerns regarding the long-term availability of these critical resources.^3,4^ To address this challenge, innovative resource recovery techniques and improved reuse rates are critical to meeting the rising demand for valuable metals from various sources, including industrial wastewater, brine, and spent products, therefore fostering a more sustainable and circular economy.^4−7^ Among these critical metals, lithium (Li) has gained significant attention due to its indispensable role in rechargeable Li^+^-ion batteries (LIBs).^6,8,9^ The soaring demand for LIBs has widened the Li^+^ supply demand gap,^2,10^ resulting in increasing concerns about the sustainability and environmental impact of Li^+^ mining and processing.^6,11−13^ This supply shortage exacerbates environmental and social issues, including increased greenhouse gas emissions, water scarcity, and displacement of local communities.^14^ Consequently, developing efficient, selective, and environmentally benign separation technologies for extracting Li^+^ from various sources is imperative to ensure a sustainable and resilient Li^+^ supply chain.^1,3,11^

Salt-lake brines have emerged as the most viable source of Li^+^, with concentrations ranging from 200 to 4000 mg/L.^12,15^ Apart from Li^+^, these brines, typically found in arid regions, also contain a substantial amount of sodium (Na^+^), potassium (K^+^), calcium (Ca^2+^), and magnesium (Mg^2+^), along with other interfering ions. Traditional Li^+^ mining from brines relies on evaporation-precipitation methods, leveraging solar energy and wind to concentrate Li^+^ salts.^10,16^ While this approach proves to be cost-effective and profitable, it suffers from slow processing times (1–2 years from brine pumping to lithium carbonate (Li_2_CO_3_) production), high freshwater consumption (∼100–800 m^3^ per tonne of Li_2_CO_3_ produced), dependency on weather conditions, substantial waste generation, and inefficiencies in treating brines with high concentrations of interfering ions (e.g., high Mg^2+^-to-Li^+^ mass ratio (MLR)).^7,17−19^ In response to these challenges, direct Li^+^ extraction (DLE) techniques have arisen as sustainable alternatives, aiming to bypass the reliance on evaporation ponds.^7,8,20^ DLE methods encompass diverse methodologies, including adsorption,^21,22^ liquid–liquid extraction,^23,24^ electrochemical methods,^16,25^ selective precipitation,^10,15^ and membrane processes.^12,22^ Compared to conventional evaporitic methods, DLE techniques offer advantages such as shorter processing times, reduced freshwater use, and higher Li^+^ extraction efficiencies.^10,12^ Moreover, DLE techniques have the potential to minimize the environmental footprint of Li^+^ extraction by mitigating land use, greenhouse gas emissions, and waste generation. Despite the widespread adoption, DLE techniques still face challenges related to high capital costs, high energy consumption, and sustainable production issues. Substantial research and development efforts are thus needed to customize Li^+^ extraction techniques tailored to the unique composition of specific brine sources.^14,16,22,26^

**Figure 1:**
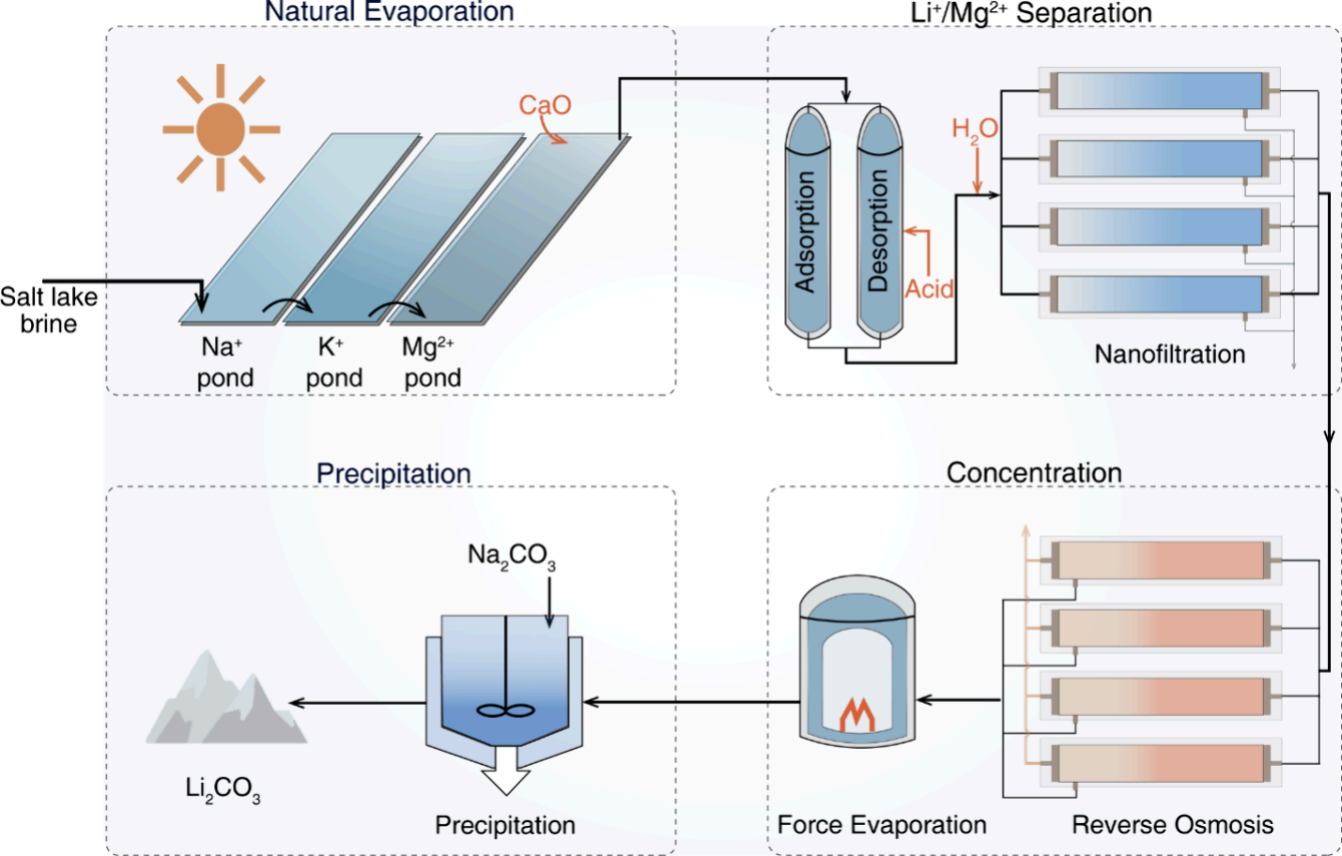
**Nanofiltration (NF)-reverse osmosis (RO) integrated treatment train for Li**^**+**^**extraction from salt-lake brines**. Salt-lake brines are characterized by high ionic strength (typically 150–300 g·L^–1^ total dissolved solids), complex composition, and high scaling potential.^12^ This process integrates NF with strategic pre- and post-treatment steps to form a complete treatment train for efficient Li^+^ extraction. Pretreatment involves the precipitation of sodium and potassium (e.g., KCl fertilizer production), partial magnesium precipitation (e.g., via the addition of CaO), and Li^+^-selective adsorption, for initial Li^+^ enrichment. The core separation stage utilizes an NF unit for separation, followed by post-treatment steps that include RO concentration of the Li^+^-enriched NF permeate, evaporation of the RO retentate, and the final precipitation for the production of Li_2_CO_3_. This integrated treatment train, leveraging the unique properties of NF membranes in conjunction with carefully designed pre- and post-treatment processes, offers a promising approach for the sustainable and cost-effective Li^+^ extraction from complex salt-lake brines, addressing the growing global demand for this critical resource. This figure was adapted with permission from ref (57) (Copyright 2024 Springer Nature).

Among DLE techniques, membrane-based separation processes have shown great potential for effectively extracting Li^+^ from salt-lake brines.^4,27−29^ For instance, the integration of nanofiltration (NF) and reverse osmosis (RO) technologies has been widely adopted for Li^+^ extraction from the brines, particularly those with high MLR (see Figure 1).^23,30,31^ A typical NF-RO integrated process for Li^+^ extraction involves the use of evaporation ponds for precipitating Na^+^ and K^+^ salts and concentrating Li^+^, followed by NF units to separate Li^+^ and interfering ions (e.g., Mg^2+^), RO to further concentrate the Li^+^-rich NF permeate, and a final precipitation step for Li_2_CO_3_ production.^32^ Despite its wide applications, this process still faces challenges due to the high costs and/or low treatment efficiency. These challenges primarily stem from the low Li^+^ purity (i.e., the mass fraction of Li^+^ in the permeate stream) and/or low Li^+^ recovery (i.e., the proportion of Li^+^ from the feed that is eventually recovered in the permeate) of the NF unit, necessitating the design of more advanced NF membranes and/or processes.

Selective solute–solute separation using NF membranes has emerged as a research frontier for advancing the Li^+^ extraction.^33,34^ For example, in a binary Li^+^ extraction system between Li^+^ and Mg^2+^, the challenges lie in the similar hydrated size of the two ions and the prevalence of high MLR in brines.^31,35^ These factors pose significant challenges in discriminating Li^+^ and Mg^2+^ using NF technology.^36,37^ To address these challenges, membrane scientists have been exploring various modification techniques to enhance Li^+^/Mg^2+^ selectivity, including regulation of charge properties,^38,39^ manipulation of monomer diffusion,^40,41^ utilization of new monomers,^42,43^ incorporation of additives,^44,45^ post-treatment methods,^36,46^ construction of interlayer,^47,48^ modification of porous substrates,^49,50^ layer-by-layer assembly,^51,52^ and etc.^53−55^ In addition to membrane modifications, recent literature suggests that the traditional separation evaluation framework for water-solute separation, which adopts water permeance (*A*) and Li^+^/Mg^2+^ selectivity, might not be suitable for the Li^+^ extraction process. Instead, new crucial evaluation metrics include attaining high Li^+^ recovery by extracting the majority of Li^+^ from the feed stream and high Li^+^ purity by generating the permeate stream with high Li^+^ selectivity.^32^ Nevertheless, existing studies primarily analyze the separation performance of NF membranes at the small coupon scale,^56^ making the translation to system-scale applications challenging. For instance, important factors, such as membrane fouling, concentration polarization, and overall process configuration, are often overlooked.^56^ Furthermore, the economic viability of NF-based Li^+^ extraction studies warrants further exploration, including cost comparisons and energy consumption.^4^ Given these considerations, a timely and systematic review is called for to critically assess recent progress in NF separation technology for Li^+^ extraction, which could bridge the gap between the lab-scale outcomes and industrially relevant demands.

In this review, we aim to provide a comprehensive summary of recent progress in NF techniques for Li^+^ extraction from salt-lake brines, encompassing membrane modification strategies, key performance metrics, and overall process optimization. Leveraging machine learning techniques, we will quantify the impact of crucial features (e.g., membrane intrinsic properties and operational parameters) on contributing to high-performance Li^+^-selective NF membranes. Furthermore, we will conduct a system-scale analysis, considering membrane fouling, concentration polarization, and overall process configuration, aiming to bridge the gap between coupon-scale NF membrane performance and real-world applications. In addition, we will discuss the techno-economic and life-cycle assessments of Li^+^ extraction process to illuminate the economic competitiveness and environmental footprint of NF-based Li^+^ extraction. Beyond the standalone NF technology, we will examine the opportunities for an integrated system, such as combining NF with capacitive deionization (CDI) to advance the Li^+^ extraction efficiency. By identifying promising hybrid processes and future research directions, this review aims to guide future research endeavors and expedite the development of sustainable and cost-effective lithium extraction avenues.

## 2 Designing Highly Li^+^-Selective NF membrane

### 2.1 Separation Mechanisms

**Figure 2:**
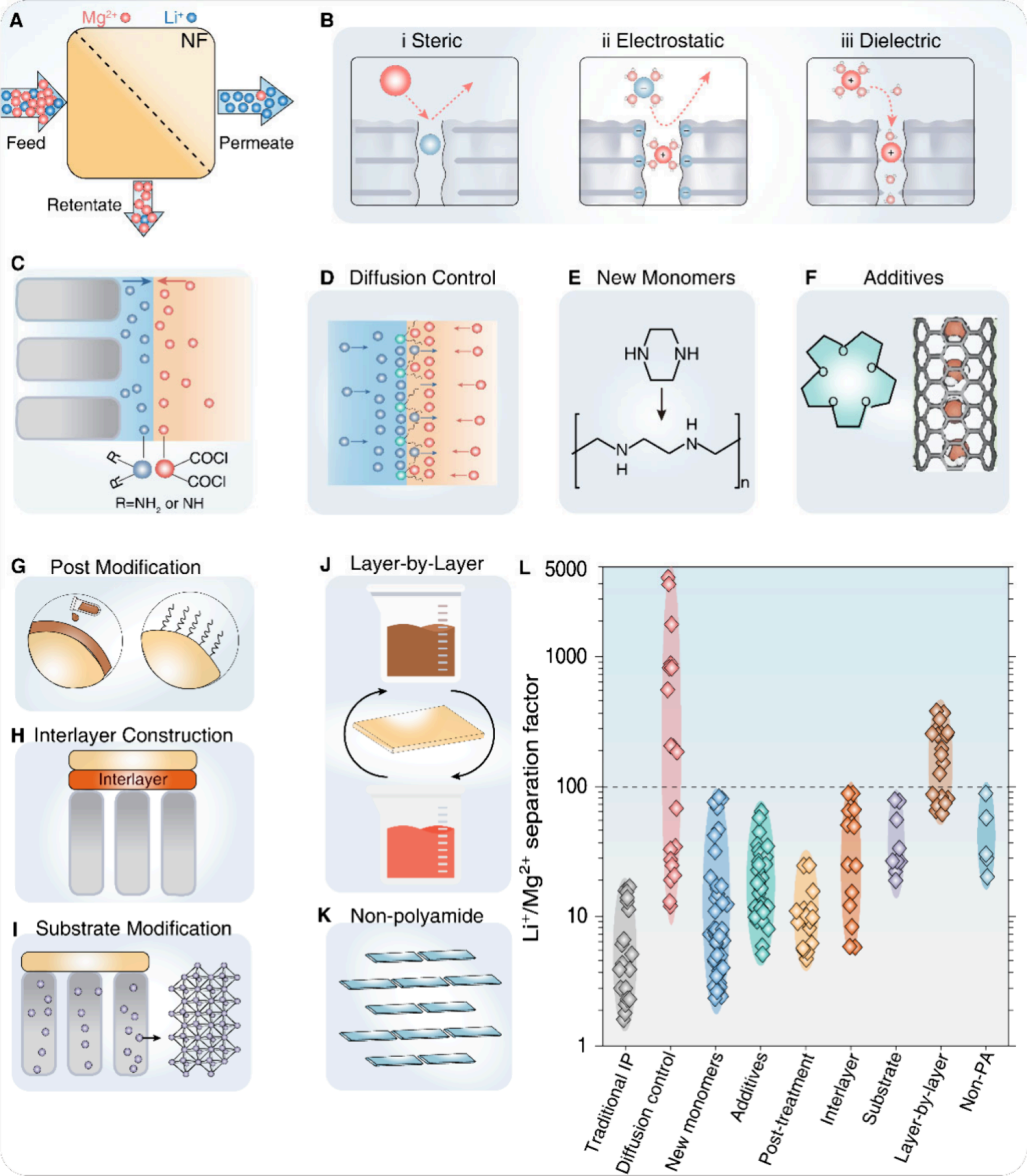
**Separation mechanisms, modification strategies, and separation performances for nanofiltration (NF) membranes in Li**^**+**^**/Mg**^**2+**^**separation**. (A) Schematic illustrations of solute–solute separation for Li^+^/Mg^2+^ separation. (B) Separation mechanisms of NF membranes, including (i) steric hindrance, (ii) Donnan exclusion, and (iii) dielectric effects. (C) Typical interfacial polymerization reaction for the fabrication of conventional NF membranes. Modification strategies for enhancing NF membranes to differentiate between Mg^2+^ and Li^+^ ions: (D) Monomer diffusion manipulation by controlling monomer diffusion rate during the IP process to optimize the physicochemical properties of the polyamide rejection layer; (E) Novel monomers by the incorporation of functional monomers to enhance Li^+^ selectivity and/or permeability; (F) Additive incorporation with the integration of molecular additives to create preferential pathways for Li^+^ transport; (G) Post modification using surface treatment or grafting to fine-tune membrane surface properties and charge; (H) Interlayer construction by the introduction of an intermediate layer between the substrate and the rejection layer to enhance separation performance; **(I)** Substrate modification by altering the properties of the support layer to improve overall membrane performance; **(J)** Layer-by-layer assembly with the precise control of membrane structure and composition through sequential deposition of polyelectrolytes or other materials; and **(K)** Nonpolyamide membrane by the exploration of alternative membrane chemistries to overcome limitations of traditional polyamide-based membranes. **(L)** Critical analysis of Li^+^/Mg^2+^ separation factors achieved by NF membranes synthesized using the modification strategies (C–K). The data demonstrate the significant improvements in selectivity achieved through these modification techniques, with some strategies yielding separation factors >4000,^37^ compared to <5 for commercial NF membranes.^32^ These strategies aim to overcome the trade-off between water permeance and Li^+^/Mg^2+^ selectivity, enabling the development of high-performance NF membranes for efficient Li^+^ extraction.

The transport of ions for NF membranes is primarily governed by three mechanisms: steric hindrance, Donnan exclusion, and dielectric effect (Figure 2B).^58,59^ These mechanisms collectively determine the membrane’s selectivity and efficacy for solute–solute separation. Steric hindrance arises when the dimensions of hydrated or bare ions approach the membrane’s pores or free-volume elements.^28^ For instance, in the context of Li^+^ extraction, this mechanism allows smaller ions to permeate through the membrane readily, while effectively rejecting larger ions. Notably, hydrated ions possess the ability to modulate their size by restructuring or shedding their hydration shell to accommodate the pore dimensions. In contrast, bare ions can only traverse the membrane if the free-volume elements are adequately large.^60,61^ For ions of similar size but differing charges (e.g., Li^+^ and Mg^2+^), the Donnan potential at the membrane-solution interface predominantly governs their transport.^62,63^ This electrochemical potential difference, resulting from the unequal distribution of ions between the membrane and solution, could cause the repulsion of co-ions (i.e., ions with the same charge as the membrane) and the attraction of counterions (i.e., ions with the opposite charge as the membrane).^64,65^ The dielectric effect plays another important role in affecting ion transport on the basis of differences in permittivity or electric polarizability between the bulk solution and the membrane phase,^28,58,61^ where the high permittivity of the bulk solution promotes ion stabilization through their hydration shells. For the Li^+^/Mg^2+^ separation, compared to Li^+^, this energy barrier is more pronounced for Mg^2+^ owing to their higher charge valence, hindering their passage through the membrane.^66,67^ Although these mechanisms operate on distinct principles, their interplay significantly influences the overall membrane separation performance.^68^ For example, strategic tuning of membrane pore size and surface charge could potentially leverage both steric and Donnan effects to achieve high Li^+^/Mg^2+^ selectivity while maintaining high Li^+^ permeability.^36,46^ Future studies should explore the synergistic interactions of these mechanisms to achieve precise Li^+^/Mg^2+^ separation from complex, highly saline brine solutions.^53,65^ Such investigations could pave the way for effective membrane designs that optimize Li^+^ extraction efficiency in diverse operational conditions.

### 2.2 Modification Strategies

The development of highly Li^+^-selective NF membranes is of paramount importance for optimizing Li^+^ extraction in NF-based separation processes. Ideally, these membranes should allow unimpeded Li^+^ passage while achieving nearly complete rejection of interfering ions (e.g., Mg^2+^). In assessing NF membranes performance for Li^+^/Mg^2+^ separation, researchers commonly employ the separation factor (*SF*_Li/Mg_), defined as the ratio of Li^+^ passage to that of Mg^2+^.^36^ Higher *SF*_Li/Mg_ values indicate superior Li^+^/Mg^2+^ separation efficiency, offering several operational advantages, including streamlined pre- and post-treatment steps for Li^+^ purification, enhanced Li^+^ purity in NF permeate stream, and overall cost reductions (e.g., lower chemical consumption and reduced footprints).^7^ Recent literature suggests that high water permeance is another critical performance indicator for optimizing Li^+^ extraction processes,^30^ which could lead to lower operating pressures, thereby reducing energy consumption, or yield cost saving by requiring less total membrane area.^69^ Despite these potential benefits, NF-based Li^+^ extraction processes face an intrinsic trade-off between selectivity and water permeance. This fundamental challenge manifests in two primary scenarios: (1) membranes exhibiting high Li^+^ selectivity often correlate with diminished water permeance, impeding high process throughput and escalating energy consumption; (2) higher water permeance typically results in lower Li^+^ selectivities, necessitating additional downstream purification steps to achieve desired Li^+^ purity.

Another critical challenge for Li^+^ extraction from salt-lake brines is the complex feedstock matrix characterized by high concentrations of ions and organic matter. This complexity can adversely affect the long-term stability and increase the fouling propensity of NF membranes.^31,35,70^ Furthermore, the scalability and cost-effectiveness of membrane fabrication methods emerge as a paramount consideration for large-scale industrial implementation, requiring further optimization to ensure the economic viability of NF-based Li^+^ extraction processes.^4,46^ To address these challenges, researchers have devoted extensive efforts to exploring a diverse range of strategies targeting specific aspects of membrane design, synthesis, and fabrication.^38,53^ This section provides a critical review of prevailing modification strategies for NF membranes, including charge property regulation, monomer diffusion manipulation, new monomer adoption, additives incorporation, postmodification techniques, interlayer construction, layer-by-layer (LBL) assembly, and development of nonpolyamide membranes. To offer critical insights into the efficacy of these strategies, we conducted a statistical analysis comparing the Li^+^/Mg^2+^ selectivity achieved through these strategies to that of conventional membranes (see Figure 2L). By critically examining these strategies through the lens of both fundamental science and practical application, we aim to bridge the gap between laboratory innovations and industrial implementation. This approach aligns with the overarching goal of accelerating the development of sustainable, efficient, and economically viable Li^+^ extraction processes.^14^

#### 2.2.1 Charge Property Regulation

The evolution of NF membranes for Li^+^ extraction has been marked by incremental advancements since their initial application in 2006, which yielded a modest Li^+^/Mg^2+^ separation factor (*SF*_Li/Mg_) of approximately 3.5 using a commercial NF membrane.^71,72^ Subsequent investigations into various commercial membranes, including Desal DK,^73−76^ Desal DL-2540,^74^ DK-1812,^77^ and NF90 membranes^78^ aimed to enhance Li^+^ extraction efficiency. However, these membranes face inherent limitations of negatively charged rejection layer (i.e., less pronounced Donnan exclusion effect for rejecting positively charged interfering ions), a consequence of the hydrolysis of acyl chloride groups during the interfacial polymerization (IP) reaction.^28,34,79,80^ Consequently, these commercial membranes often exhibit low *SF*_Li/Mg_ values (<5) and moderate water permeance (e.g., ∼10 L·m^–2^·h^–1^·bar^–1^),^30,71,75^ which is less favorable for achieving a highly efficient Li^+^ extraction process. To overcome these constraints, researchers have shifted their attention to developing positively charged NF membranes. These membranes leverage enhanced electrostatic repulsion force toward Mg^2+^ compared to Li^+^, thereby augmenting the Li^+^/Mg^2+^ separation efficiency.^36,81^ This novel strategy typically involves: (1) integration of positively charged monomers;^82^ (2) incorporation of functional groups (e.g., amines or quaternary ammonium moieties^83,84^) within the membrane matrix; and (3) surface modification of existing membranes to impart positive charge.^85^ A notable example is the use of polyethylenimine (PEI). Leveraging its abundant amine groups, PEI-modified membranes have demonstrated significant improvements in Li^+^/Mg^2+^ separation.^38,39,53,82,83,86−92^ However, the charge regulation strategy presents a critical trade-off: while the incorporation of positively charged groups leads to enhanced Mg^2+^ rejection, it could also result in a more compact polyamide (PA) rejection layer due to the increased charge density,^36^ thus impeding water permeance. Future research should focus on the deliberate manipulation of charge density and PA layer cross-linking degree to achieve an optimal balance between high water permeance and high Li^+^ selectivity in the extraction process.^36,84,93^

#### 2.2.2 Monomer Diffusion Manipulation

The membrane rejection layer, predominantly composed of PA chemistry (Figure 2C), is the core component of NF membranes.^86^ To date, IP reaction dominates the avenue for fabricating this crucial rejection layer.^94^ However, the conventional IP process presents inherent limitations, including rapid reaction kinetics and self-limiting nature characterized by multiscale heterogeneity and nonuniform pore sizes distribution.^27,68^ These factors pose grand challenges to achieving precise Li^+^/Mg^2+^ separation.^28,68^ To overcome this hurdle, researchers have been exploring various strategies to modulate monomer diffusion and interfacial enrichment at the water/oil interface during the IP process (Figure 2D).^27,41,95^ These approaches aim to enable finer control over the structure and properties of the PA layer, thus greatly augmenting the Li^+^/Mg^2+^ separation efficiency. A comprehensive statistical analysis of the literature (Figure 2L and Supporting Information S3) reveals that monomer diffusion control appears to be the most effective approach (Figure 2L). This superiority is attributed to the optimized IP reaction and the resultant homogeneity of pore distribution in the PA rejection layer.

One promising avenue involves the incorporation of surfactant molecules, such as oil-soluble dodecyl phosphate (DDP)^37^ and water-soluble benzyltributylammonium chloride (BtBAC).^96^ These surfactants could self-assemble at the oil/water interface, influencing amine monomer diffusion and regulating membrane pore structure.^27,68^ The resultant membranes exhibit well-homogenized pore sizes, ensuring that membrane pore size is smaller than that of hydrated Mg^2+^ ions yet larger than dehydrated Li^+^. This precise pore size control facilitates ultrahigh Mg^2+^ rejection (up to 99.96%) and ultrahigh Li^+^/Mg^2+^ selectivity (up to 4147).^37^ Beyond surfactant engineering, researchers are exploring different strategies to mitigate the intrinsic heterogeneity of the PA layer, including reversed interfacial polymerization (RIP),^91,97^ gas–liquid interface IP process (e.g., evaporating amine aqueous solution to amine gas),^98^ strategic solvent selection (e.g., transitioning from hexane to xylene),^99^ and decoupled bulk/interfacial diffusion (e.g., increasing viscosity of amine aqueous solution by adding ionic liquid).^40^ These methods effectively manipulate monomer interfacial diffusion and tailor the PA layer structure, resulting in membranes with superior Li^+^/Mg^2+^ separation capabilities. Despite these advancements, challenges lie in achieving membrane scalability, reproducibility, and long-term stability.^28,53^ Future studies should not only focus on manipulating the IP reaction at the molecule level but also explore the key parameters for achieving large-scale membrane production.

#### 2.2.3 New Monomer Adoption

The development of high-performance NF membranes for efficient Li^+^ extraction is inherently linked to the rational selection and adoption of novel monomers (Figure 2E). These monomers, characterized by unique functional groups, distorted geometry, and charge properties, serve as building blocks for the PA layer, opening avenues for researchers to engineer the membranes’ physicochemical properties and optimize their Li^+^/Mg^2+^ separation efficiency.^100^ A promising strategy is the utilization of monomers with distorted and noncoplanar geometry, such as cyclopentane tetracarboxylic acid chloride (CPTC),^100^ quaternized-spiral piperazine (QSPIP),^54^ and Gemini-electrolyte monomer (GEM).^46^ Their incorporation favors the formation of microporous structures or free-volume elements within the membrane matrix, resulting in well-ordered nanostructures on the membrane surface and enhanced water permeance.^46^ Concurrently, positively charged amine monomers have been investigated as promising substitutes for piperazine (PIP) adopted in commercial NF membrane chemistry.^101^ These alternatives, including 1,3-diaminoguanidine hydrochloride (DAGH),^102^ polyallylamine (PAA),^103^ 1,3,5-tris(bromomethyl)benzene (TBB),^83^ 1,4-Bis(3-aminopropyl)piperazine (DAPP),^90^ and γ-cyclodextrins (CDs),^104^ leverage their inherent positive charges and the hydrophilicity of multiple amine groups to increase the Li^+^/Mg^2+^ separation efficiency and membrane water permeance.^82,102,104^ Another promising strategy involves the functionalization of existing monomers. For example, modification of PEI with 3-diamino-methyl-cyclohexyl triethoxysilane (DTES),^82^ or ethylenediamine (EDA),^39^ have yielded satisfactory separation performance,^105^ offering opportunities to fine-tune the balance between charge density and cross-linking degree in the PA layer. Furthermore, the exploration of unconventional polymers such as poly(ionic liquid)s,^106^ 1,4,7,10-Tetraazacyclododecane (TAD), and 1,2,4,5-Tetrakis(bromomethyl)benzene (TBB),^42^ holds promise for fabricating defect-free and ultrathin NF membranes with unique structural and chemical properties. Collectively, these novel monomer-based modifications demonstrate significant potential for improving membrane Li^+^/Mg^2+^ separation factor (Figure 2L), water permeance, stability, and scalability. Such advancements pave the way for the realization of efficient and sustainable Li^+^ extraction processes, offering a promising trajectory for future research and development in this field.^100^

#### 2.2.4 Additives

The incorporation of additives during the IP process is also an effective approach for engineering the physicochemical properties of the resulting PA selective layer (Figure 2F).^38^ This versatile strategy, which generally enhances the *SF*_Li/Mg_ compared to control membranes (Figure 2L), employs various types of additives, each serving specific functions to optimize membrane performance. For example, Li^+^-affinity additives, such as crown ethers,^44,107^ and cyclen,^108^ could facilitate the formation of exclusive Li^+^ transport channels within the PA matrix, thereby enhancing Li^+^ transport while hindering the passage of interfering ions (e.g., Mg^2+^). Molecular dynamics simulations have elucidated the formation of these Li^+^-selective complexes, revealing optimized binding energies and geometries that enable efficient, exclusive Li^+^ transport through membranes.^44,107,109^ Alternatively, cage-like structure additives,^110^ exemplified by amino-functionalized polyhedral oligomeric silsesquioxane (8NH_2_–POSS),^111^ generate additional water channels within the PA layer, improving water transport without compromising the *SF*_Li/Mg_. The hydrophilic nature of amino groups further enhances overall water permeance. Certain additives, such as Girard’s Reagent T (GRT),^112^ can modulate the kinetics of the IP reaction, altering the membrane’s cross-linking degree. Specifically, GRT effectively reduces the cross-linking density of the PEI-based PA layer by end-capping TMC monomers,^112^ creating a looser structure network with increased free-volume elements that facilitate water and Li^+^ passage while maintaining Mg^2+^ rejection.

Nanomaterial-based additives, such as layered double hydroxides (LDHs),^51^ hold promise in selective water channels and enhancing the hydrophilicity of the PA layer. The compatibility with the PA matrix and stability in aqueous or organic phases remains challenging.^113^ To this end, researchers have pregrafted compatible functional groups onto nanomaterials, such as potassium carboxylate functionalized multiwall carbon nanotubes (MWCNTs-COOK),^113^ hydroxyl contained multiwalled carbon nanotubes (MWCNTs–OH),^89^ aminated graphene quantum dots (GQDs-NH_2_),^114,115^ zwitterion-carbon nitride (BHC–CN),^70^ and amine-functionalized carbon dots (Am-CDs).^116^ These functionalized nanomaterials exhibit improved dispersibility and stability within the PA matrix.^117^ To further improve their stability, cross-linking agents (e.g., glutaraldehyde (GA)^51^) have been employed to form covalent bonds between the incorporated nanomaterials and the PA matrix, preventing potential leaching during long-term operation and maintaining membrane structural integrity.^118^ Metal–organic frameworks (MOFs) represent another promising class of nanomaterial additives. Pregrafted functional groups in MOFs such as NH_2_-MIL-101(Cr)^47^ and UiO-66-NH_2_^119^ could provide rapid transport pathways for Li^+^ in the resulting NF membranes. The well-defined pore structures and high surface areas of MOFs,^120^ combined with -NH_2_ groups that exhibit favorable interactions with Li^+^, collectively facilitate the rapid passage of Li^+^ through the membrane while effectively rejecting Mg^2+^.^47,119^

While achieving uniform and stable dispersion and compatibility of additives within the PA matrix is crucial for ensuring reliable separation performance, future studies should focus on in-depth mechanistic investigations adopting advanced characterization techniques (e.g., high-resolution transmission electron microscopy (HRTEM)^121^ and three-dimensional (3D) tomography).^122,123^ These advanced characterization techniques can provide valuable insights into the dispersion and interaction of nanomaterials within the PA layer, guiding the optimization of the IP-based NF membranes for targeted Li^+^/Mg^2+^ separation. Additionally, long-term stability assessment under realistic industrial conditions is another important factor in validating the durability and reliability of membrane separation performances. It is worthwhile to note that the incorporation of nanomaterials in NF membranes may incur concerns regarding the potential leakage, presenting challenges encompassing environmental, health, and operational risks.^124^ Mitigation strategies include novel grafting techniques and surface modifications to improve polymer-nanomaterial interactions. The development of nontoxic nanomaterials presents an innovative approach to maintaining functionality even if nanomaterials are leaked. Future research should prioritize comprehensive life cycle assessments, standardized protocols for evaluating nanomaterial leakage, and *in silico* models to predict nanoparticle–membrane interactions.^125,126^

#### 2.2.5 Postmodification

Postmodification techniques present a powerful strategy to enhance the separation efficiency of NF membranes while preserving the structural integrity of the PA layer.^112^ These methods enable precise manipulation of membrane surface or bulk properties, rendering fine-tuning of surface charge, hydrophilicity, and pore size for achieving superior Li^+^/Mg^2+^ separation performance (Figure 2L). A notable approach is the secondary IP process. This method introduces a high density of reactive amine groups onto the nascent PA selective layer,^36,53^ effectively augmenting the membrane’s surface charge through bonding with the residual acyl chloride groups. The resultant increase in electrostatic repulsion force markedly enhances Mg^2+^ rejection. Following this strategy, researchers have explored a diverse array of amino-containing modifiers for the secondary IP process, including quaternary ammonium salts,^84,127^ ionic liquids,^36,93,128−132^ and amino-rich polymers^133−137^ for the second IP process. For instance, quaternary ammonium salts with different alkyl chain lengths could enable precise control over the charge density and hydrophobicity of the modified surface, while ionic liquids with specific anions and cations can be strategically selected to enhance the Li^+^ selectivity and water permeance.^84,128,138^

The principles of coordination chemistry have also been harnessed for membrane modification, exemplified by *p*-aminosalicylic acid-Fe(III) chelation^139^ and ethylenediaminetetraacetic acid (EDTA) grafting.^88^ These methods introduce metal–ligand complexes onto the membrane surface, which selectively sequester Mg^2+^, thereby enhancing its rejection through size exclusion and electrostatic interactions. Another intriguing approach that has gained attention is the swelling-embedding-shrinking strategy. This method employs carefully selected solvents to temporarily expand the PA matrix, facilitating the intercalation of modifiers such as histamine^140^ and diethylenetriamine (DETA),^141^ which enables precise manipulation of membrane pore size to optimize Li^+^/Mg^2+^ selectivity (see Separation Mechanism in Section 2.1 for further details). Through careful selection of the swelling solvents and modifiers, researchers are able to fine-tune the pore size distribution of the membrane to achieve optimal Li^+^ permeability while preventing Mg^2+^ from passing through. However, the long-term stability of modified membranes under extreme environments remains a significant challenge.^142^ Exposure to high salinity, extreme pH, and organic foulants can potentially lead to the degradation or detachment of modified layers, compromising separation performances.^131,143^ To address this issue, research efforts have been directed toward developing novel functional materials with enhanced compatibility, stability, and resistance to harsh environments.^144^ A promising approach involves the covalent grafting methods, which establish strong chemical bonds between the modifiers and the membrane surface, improving the stability of the modified layer. Notable examples include the utilization of silane coupling agents with reactive functional groups containing amino or epoxy moieties.^145,146^ Another strategy is the development of self-healing materials capable of *in situ* repair of the modified layer during operation.^147^ This approach incorporates reversible bonds, such as dynamic covalent bonds or supramolecular interactions, which can break and reform in response to external stimuli like pH changes or light irradiation.^147,148^

#### 2.2.6 Interlayer Construction

The construction of an interlayer between the rejection layer and the supporting substrate presents an advanced strategy for enhancing the Li^+^/Mg^2+^ separation of NF membranes (Figure 2H and L).^47,149^ This approach leverages the interlayer as a multifunctional platform, influencing various aspects of membrane performance. An important mechanism of interlayers lies in their ability to regulate the IP process. Specifically, an interlayer could act as a barrier during the IP process, preventing the formation of PA intrusion in the substrate pores.^150^ This interlayer-enhanced membrane could benefit from (1) enhanced overall membrane permeance, facilitating a faster and more efficient extraction process^151^ and (2) the creation of a more uniform surface for the PA selective layer with reduced defects,^152^ which, in turn, improves separation efficiency given that fewer unwanted ions can pass through these defects (e.g., enhanced Mg^2+^ rejection during the Li^+^/Mg^2+^ separation). The judicious selection of interlayer materials can impart additional functionality to the overall membrane structure.^48^ For instance, porous organic polymers (POPs),^150,153^ engineered with high affinity for Li^+^ ions, can be adopted to construct interlayers that selectively capture Li^+^, leading to improved Mg^2+^ rejection and ultimately, enhanced Li^+^/Mg^2+^ selectivity. Another avenue explores the use of positively charged interlayers, which not only regulate the pore size of the overall membrane but also increase its surface charge density, further enhancing separation performance.^48,154^

The interlayered approach also offers the benefit of the gutter effect, where the transport length of solvent and solutes can be redirected to control their overall transport resistance.^155^ Previous research efforts have primarily focused on water-solute separation, where the incorporation of a highly permeable interlayer could greatly reduce the overall resistance of water transport by reducing the transport resistance in the transverse direction. Such resistance reduction could translate into up to an order of magnitude higher water permeance and result in up to ∼80% savings on energy consumption in water purification.^151^ Beyond water purification, the effectiveness of the gutter effect for solute–solute separation (e.g., Li^+^/Mg^2+^ separation) has been far less discussed in the literature. Opportunities lie in the transport resistance control for manipulating the overall solute–solute selectivity of the membrane based on the interlayer approach.^156,157^ For example, the introduction of a Li^+^-selective interlayer could not only improve the Li^+^ transport in the interlayer itself but also reduce its transport resistance in the top dense rejection layer. As a result, the overall transport resistance can be greatly reduced. With a high Mg^2+^ rejection maintained, the overall Li^+^/Mg^2+^ selectivity of the membrane can be significantly improved. Future studies should explore the untapped opportunities regarding the interlayer-optimized solute–solute separation.^151^

#### 2.2.7 Substrate Modification

Optimizing the porous substrate is a promising approach to enhance the overall membrane separation performance toward a more efficient Li^+^ extraction process (Figure 2I,L).^49^ To strengthen the interaction between the porous substrate and the top rejection layer, hydrophilic additives or nanomaterials, such as polyvinylpyrrolidone (PVP),^158^ graphene oxide (GO)^159^ and two-dimensional (2D) MXene nanosheets,^50^ can be incorporated to prepare mixed-matrix substrates. These materials tailor the physicochemical properties of the substrate, followed by the IP reaction to obtain the final NF membrane. Acting as bridges, these additives or nanofillers could strengthen the interfacial adhesion between the substrate and the upper PA layer, resulting in enhanced membrane structural stability,^47^ water permeance,^150^ and selectivity.^160^ Additionally, modifying substrate surface properties, using polyelectrolytes^160^ or hyperbranched polymers^161^ as the modifiers, could promote more extensive reactions between the substrate and the monomers during the IP process. This results in a denser structure and reduces the negative charge of the PA layer, enhancing Mg^2+^ rejection.

A critical challenge in the Li^+^ extraction process is how to mitigate membrane fouling and concentration polarization (CP), phenomena that significantly impair membrane separation performance.^32,162^ Research efforts have been focusing on engineering substrates with enhanced surface roughness, including crumpled surface^163,164^ or surface patterns.^165^ Specifically, a rougher surface may potentially generate localized turbulence to mitigate localized fouling and/or localized CP issues.^163^ For the Li^+^/Mg^2+^ separation, given that larger amounts of Mg^2+^ could be retained on the membrane surface compared to that of Li^+^ due to its larger hydrated size and higher valence (refer to Section 2.1), the mitigated fouling and CP could be more pronounced in reducing Mg^2+^ accumulation on membrane surface to enhance its rejection. In contrast, this effect is less significant for Li^+^, thereby significantly improving the overall Li^+^/Mg^2+^ selectivity. Moreover, the rate of membrane fouling and CP issues can be exponentially increased with the enhanced water flux.^32,162^ While traditional wisdom often adopts the observed macroscopic water flux based on membrane coupons/modules, the microscopic/nanoscale water flux has been far less discussed, which could be more sensitive to membrane fouling and CP issues. Given that the macroscopic water flux is often fixed at a given application scenario, adopting a rougher membrane with enhanced surface areas may, in turn, reduce its localized water flux compared to a smoother counterpart.^166^ This reduction could be beneficial to reduce the fouling and CP effect of the overall membrane, and thus, the accumulations of different solutes on the membrane surface could be mitigated. Nevertheless, future studies should establish a systematic framework and explore the effect of substrate morphologies on solute–solute selectivity (e.g., Li^+^/Mg^2+^ separation).

#### 2.2.8 Layer-by-Layer Assembly

Layer-by-layer (LBL) assembly of the membrane rejection layer is another facile and cost-effective method for enhancing the efficiency of the Li^+^ extraction process. This technique leverages a spectrum of intermolecular interactions, including electrostatic attraction,^167^ covalent bonding,^52,168^ hydrogen bonding, and etc. (Figure 2J),^169,170^ offering effective control over membrane nanoarchitecture at the molecular level. The LBL approach has several distinct advantages, including precise control of rejection layer thickness in nanoscale, facile manipulation of surface charge through strategic selection of the outermost layer, and exquisite control over surface roughness. For example, the sequential deposition of oppositely charged polyelectrolytes enables the construction of nanoscale selective layers with tailored properties.^170^ Commonly employed polyelectrolyte pairs, such as poly(sodium 4-styrenesulfonate) (PSS) with poly(allylamine hydrochloride) (PAH),^171^ PSS/poly(diallyldimethylammonium chloride) PDADMAC,^172^ and PSS/PEI,^173^ offer diverse choices. By manipulating the number of deposition cycles, the thickness of the rejection layer and membrane properties can be fine-tuned. For instance, with the outmost layer composed of a positively charged PAH layer,^171^ the Li^+^/Mg^2+^ selectivity can be greatly improved compared to a negatively charged counterpart (Figure 2L). However, the stability of electrostatically assembled layers under harsh operational conditions—including strong acids, bases, or high salinity environments—presents a daunting challenge. Strategies to enhance membrane robustness, such as chemical cross-linking or thermal treatment,^38^ are crucial for ensuring consistent performance across diverse operational scenarios. These stabilization techniques should be carefully optimized to maintain the subtle balance between structural integrity and membrane separation efficiency.

Covalent LBL, achieved through chemical cross-linking or direct chemical bonding, offers a promising alternative, leading to membranes with enhanced stability and denser structures. For example, stacking porous organic cages (POCs) through amidation reactions could yield membranes with confined pores for the entry of Li^+^ and the interception of Mg^2+^.^174^ This approach demonstrated remarkable Li^+^/Mg^2+^ selectivity, with *SF*_Li/Mg_ reaching 64.7, highlighting the potential for molecular-level design in achieving excellent separation performance. Future endeavors of LBL assembly in the Li^+^ extraction process should explore several key areas: (1) exploration of novel nanomaterials with tailored dimensions, structures, geometries, functionalities, and porosities for LBL construction, aiming to optimize Li^+^/Mg^2+^ selectivity while maintaining high flux rates; (2) integration of data-driven approaches, such as machine learning algorithms, with mechanistic models to predict and optimize the transport behavior of LBL membranes based on their physicochemical properties; and (3) investigation of stimuli-responsive LBL systems that can adapt to changing feed compositions or operational parameters, potentially revolutionizing the efficiency and selectivity of the Li^+^ extraction processes. These interdisciplinary approaches, combining materials science, physical chemistry, and data analytics, hold the promise to advance the development of highly efficient, selective, and robust LBL membranes specifically tailored for the Li^+^ extraction from complex brine solutions.

#### 2.2.9 Nonpolyamide Membrane

While PA chemistry has long dominated commercial NF membrane fabrication, its historical optimization for seawater desalination and brackish water treatment^95,175^ has left it inadequately suited for the specific demands of Li^+^/Mg^2+^ separation, particularly in achieving high Li^+^ passage along with high Mg^2+^ rejection.^40,55^ This limitation has catalyzed an innovative shift toward alternative rejection layer chemistries, aiming to transcend the performance boundaries of traditional PA-based thin-film composite (TFC) membranes (Figure 2K). An intriguing approach involves the development of the *m*-phenylenediamine (MPD) self-polymerized TFC membrane, triggered by Cu^2+^ ions. This novel membrane exhibits remarkable adaptability in its structure and separation performance, modulated by environmental pH conditions in response to Cu-MPD complexes.^176^ The resultant membrane exhibits an *SF*_Li/Mg_ value exceeding 8, while maintaining moderate water permeance (Figure 2L). This pH-responsive behavior represents a significant advancement, potentially allowing for dynamic optimization of membrane performance in response to varying feed compositions—a crucial advantage in the complex and variable environment of brine processing. Another strategy employs electric field-assisted fabrication to construct a positively charged NF membrane. This approach strategically promotes the complexion of Mg^2+^ on the negatively charged membrane surface with polarized surface groups, such as −COOH.^177^ Alternatively, the assembly of covalent organic framework (COF) sheets into defect-free and oriented membranes, demonstrating simultaneously enhanced water permeance and Li^+^/Mg^2+^ separation efficiency, presents a shift in membrane design.^178^ Furthermore, polyester NF membranes, synthesized by incorporating novel monomers featuring “hydroxyl-ammonium” entities, signify another direction in non-PA membrane development. These membranes exhibit dense structures and positive charge, augmenting their efficacy in Li^+^/Mg^2+^ separation.^179^ Anticipating the growing demand for high-Li^+^-selective membranes, ongoing research endeavors are poised to unveil additional non-PA strategies, further diversifying the landscape of membrane technologies for Li^+^/Mg^2+^ separation.

### 2.3 Identifying the Key Parameters by Machine Learning

While tremendous efforts have been directed toward membrane materials optimization for achieving a highly efficient Li^+^ extraction process, conventional modification techniques largely rely on a trial-and-error Edisonian approach, which is relatively unreliable, time-consuming, and often ineffective.^180^ To transcend the limitations of conventional approaches and gain in-depth insights, we leveraged machine learning (ML) techniques to identify the influential features dictating *SF*_Li/Mg_ in the NF-based Li^+^ extraction process. Our analysis encompassed both membrane intrinsic properties (e.g., water permeance, molecular weight cutoff (MWCO), pore size, and surface charges) and operational parameters (e.g., pressure, water flux, feed MLR, and salt concentration). Specifically, we adopted the XGBoost algorithm to train ML models, thanks to its versatility and popularity in complex data analysis (more details can be found in Supporting Information S1).^181,182^ To mitigate potential data leakage, where the same data might be inadvertently used across testing, training, and validation phases, we partitioned the data set collected from the literature into three distinct subsets for testing, training, and validation, respectively. This partitioning was performed randomly across five random experiments, employing a 5-fold nested cross-validation approach. The outer fold consisted of five partitions, each further subjected to an inner 5-fold cross-validation. Additionally, Bayesian optimization^183^ was utilized in each random experiment to fine-tune the hyperparameters of XGBoost models, ensuring optimal performance. To interpret the ML model results, the SHapley Additive Explanations (SHAP) method^182^ was applied to elucidate the contribution of input variables (e.g., membrane physicochemical properties and operational conditions) to the predicted outcome (i.e., *SF*_Li/Mg_).

**Figure 3:**
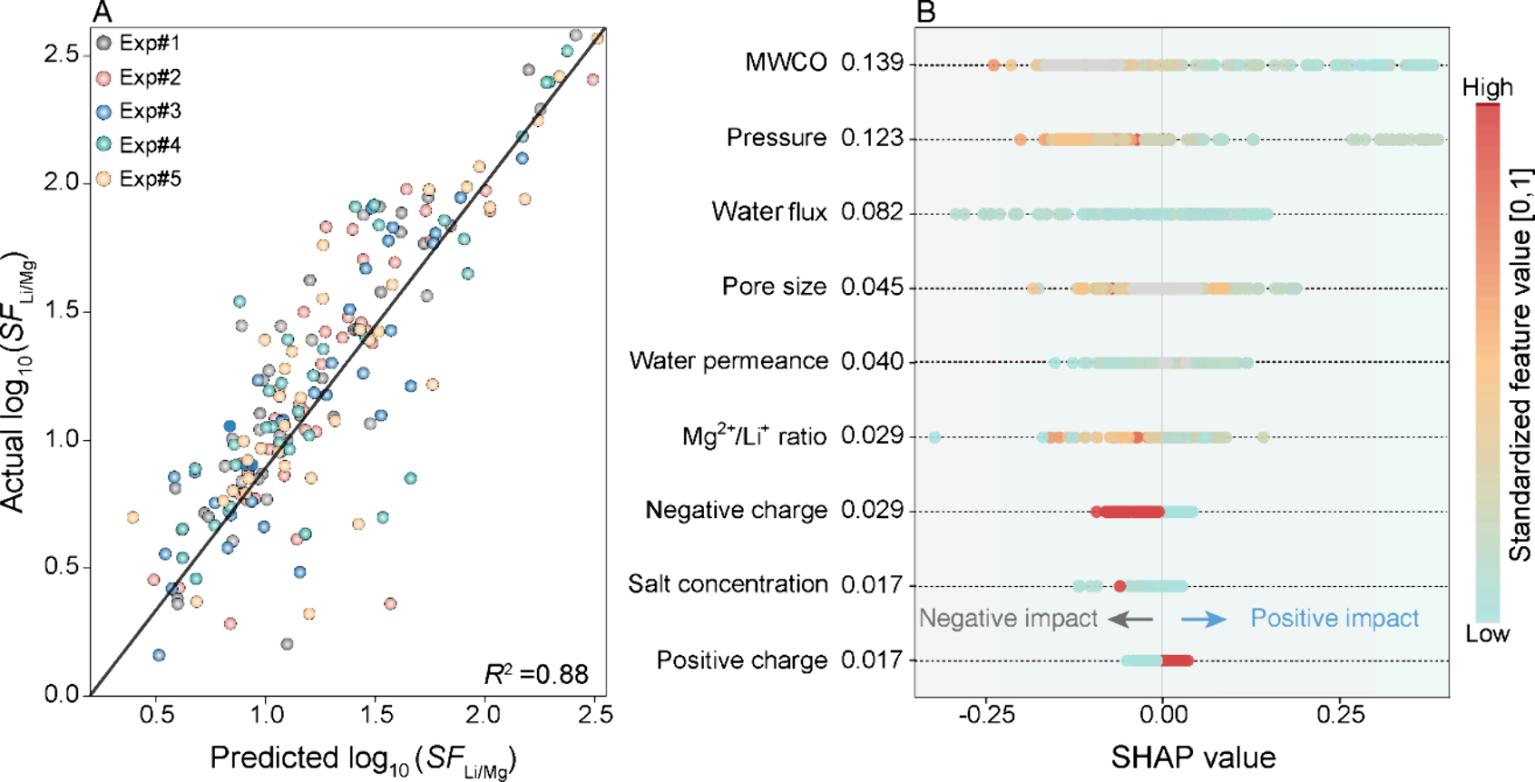
**Machine learning (ML)-assisted analysis for identifying key features of NF membrane for determining the Li**^**+**^**/Mg**^**2+**^**separation factor**. (A) Predictive performance of membrane *SF*_Li/Mg_ (log_10_*SF*_Li/Mg_) with *R*^2^ value of 0.88 using XGBoost models. A stratified nested cross-validation method (based on the outcome) based on five experiments is adopted for data training using 80% of the data points and test using 20% of the data points. The *R*^2^ value is calculated on the original scale of the data, prior to applying the log_10_ transformation. (B) SHapley Additive Explanations (SHAP) interpretation of an XGBoost model for determining membrane *SF*_Li/Mg_. The *X*-axes are the SHAP value for all features. For SHAP values >0, the larger number represents more contributions on predicted membrane *SF*_Li/Mg_. For SHAP values <0, more negative values signify a progressively negative impact. The *Y*-axes are the identified most influential features, including membrane intrinsic properties (e.g., water permeance, MWCO, pore size, and surface charges) and operational parameters (e.g., pressure, water flux, and salt concentration). The number next to each feature stands for the mean absolute SHAP value for the feature, and a larger value indicates its higher impact on the predicted *SF*_Li/Mg_ value. The feature values of data are colored, standing for the standardized feature value ranging from 0 to 1. For features with numerical values (MWCO, pressure, water flux, etc.), standardized feature values are proportional to their raw feature value. For features based on the event (e.g., negative charge or positive charge), a standardized feature value of 1 stands for the occurrence of the event, whereas 0 stands for the nonoccurrence of the event. The data for training the model is available in the Supporting Information S3.

Figure 3A demonstrates a relatively good agreement between the predicted *SF*_Li/Mg_ and actual results from the literature, with an *R*^2^ value of 0.88 for the test data sets across the five experiments. This reasonable agreement allows for further useful analysis of key features affecting the *SF*_Li/Mg_ value. Figure 3B illustrates the SHAP values of the features in experiment #1, revealing that membrane MWCO emerged as the highest mean absolute SHAP value—the most influential factor in determining the *SF*_Li/Mg_ value. This finding aligns with the importance of membrane pore size, which ranked fourth among all variables. The SHAP values further reveal a negative correlation between MWCO and *SF*_Li/Mg_ value, suggesting that a larger MWCO value (typically associated with larger membrane pores) could lead to a decreased *SF*_Li/Mg_ value (Figure 3B). This observation corroborates the size exclusion mechanisms discussed in Section 2.1, underscoring the importance of designing an NF membrane with appropriate average pore size between hydrated Li^+^ ions and Mg^2+^ ions for optimized *SF*_Li/Mg_ in the Li^+^ extraction process.^37^ As demonstrated in Section 2.2.2 and Figure 2L, the monomer diffusion-controlled approach is promising to narrow the window of the pore size distribution in NF membranes,^37^ yielding the highest predicted *SF*_Li/Mg_ value enhancement compared to other approaches. Membrane charge properties also play important roles in influencing the predicted *SF*_Li/Mg_ value. The ML results suggest that positively charged NF membranes (i.e., the nonoccurrence of the “Negative charge” feature and occurrence of the “Positive charge” feature) generally obtain higher predicted *SF*_Li/Mg_ value, aligning with the discussions in Section 2.2.1. In addition, operational parameters, including applied pressure, water flux (which can also be affected by operational pressure), feed MLR, and salt concentration, showed substantial impacts on the predicted *SF*_Li/Mg_ value. These predictions accord with the analysis from the recent literature,^32^ highlighting the critical role of appropriate water flux (e.g., ∼ 20 L·m^–2^·h^–1^) in maintaining high Li^+^/Mg^2+^ selectivity. Indeed, higher flux could result in more severe concentration polarization (CP), while low flux could lead to the weakened dilution effect, both potentially reducing salt rejection as we will discuss later in Section 3.3. Since the *SF*_Li/Mg_ value is far more sensitive to Mg^2+^ rejection compared to that of Li^+^ (will shortly be discussed in Section 3.1), the reduction in Mg^2+^ rejection could be detrimental to achieving a high *SF*_Li/Mg_ value. Accordingly, future studies need to carefully manipulate their operational parameters during the NF-based Li^+^ extraction process.

While our ML predictions generally align with the literature, we recommend researchers and practitioners take extra caution regarding potential uncertainties in the current ML models. For instance, factors like membrane water permeance, feed MLR, and salt concentration, though appearing to be less influential in dominating *SF*_Li/Mg_ value compared to other variables, can be the decisive factors in certain scenarios.^32^ In addition, it is important to understand the uncertainties and assumptions inherent in SHAP methods, among other ML explainability methods.^184^ Future work should focus on refining ML techniques to improve their accuracy and reliability. Alternatively, the current ML analysis can serve as a “virtual lab”, stimulating further experimental validations at lab or system scale to corroborate features’ importance. Moreover, the adoption of high-quality databases is paramount in ensuring the reliability and accuracy of ML-generated results.^185,186^ One promising example in this direction is the recently launched Open membrane database (OMD), a centralized archive of RO membranes for desalination and water treatment,^187^ and NF membranes for organic solvent filtration.^188^ To minimize potential erroneous information, all data and information (e.g., membrane physicochemical properties and operational parameters) uploaded to the OMD undergo careful review by an international collaboration, adhering to the FAIR principles—Findable, Accessible, Interoperable, and Reusable.^189^ Future investigations should continue to refine these ML techniques, expand high-quality databases, and validate predictions through rigorous experimental work. Another promising direction could involve the development of hybrid models that combine XGBoost with other machine learning algorithms, such as neural networks. This could enhance predictive accuracy and offer deeper insights into complex, nonlinear relationships between variables. Future research could explore the integration of dynamic modeling techniques with XGBoost to account for temporal variations in membrane performance. This approach could provide a more in-depth understanding of how factors influencing Li^+^ extraction efficiency evolve over time.

## 3 Performance Evaluation Metrics

In addition to membrane material advancement and important feature identification, membrane performance evaluation frameworks play an important role in guiding the rational design of suitable NF membranes for Li^+^ extraction. Conventional performance evaluation framework, rooted in water-solute separation metrics such as water permeance and water-solute selectivity,^190^ has proven inadequate in capturing the performance requirements of Li^+^ extraction processes.^32^ This section embarks on a critical examination of evaluation frameworks, aiming to establish a more robust and relevant set of metrics tailored to the unique challenges of selective ion separation in Li^+^ recovery. Furthermore, other evaluation frameworks proposed recently based on selective solute–solute separation will be compared.^32^ Last, we introduce a comprehensive system-level framework that caters for the real-world Li^+^ extraction process. The profound insights gained from this analysis will not only help to deepen the understanding of the performance metrics influencing the efficiency and selectivity of NF membranes in lithium extraction but also guide the development of novel membrane materials and process optimization strategies, unlocking new opportunities for sustainable Li^+^ extraction processes.

### 3.1 Existing Performance Metrics

The conventional framework for evaluating NF membrane performance primarily focuses on the membrane water permeance (e.g., the ease with which water passes through the membrane) and water-solute selectivity (e.g., the ability of the membrane to preferentially allow water to pass through while rejecting the solute), along with their trade-off correlations.^191^ In the context of Li^+^/Mg^2+^ separation, water permeance (*A*) and Li^+^/Mg^2+^ separation factor (*SF*_Li/Mg_) have often been adopted as the major indicators to evaluate the membrane separation performance. Specifically, water permeance, or water permeability coefficient (*A*), can be defined by
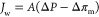
1where *J*_w_ is water flux and Δ*P* and Δπ_m_ are transmembrane pressure and osmotic pressure difference across the membrane, respectively.

For membrane rejection, the (apparent) solute rejection, *R*_i_, for a solute *i* passing through the membrane is given by
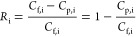
2where *C*_f,i_ and *C*_p,i_ are the solute concentrations of the solute *i* in the feed and permeate, respectively. In the context of Li^+^ extraction from salt-lake brines, another important factor, i.e., *SF*_Li/Mg_, is generally defined as the ratio of the passage of Li^+^ to Mg^2+^ through the membrane, expressed by the following equation:
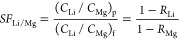
3where *C*_Li,p_ and *C*_Mg,p_ represent the concentrations of Li^+^ and Mg^2+^ in the permeate side, respectively, while *C*_Li,f_ and *C*_Mg,f_ denote their concentrations in the feed side. *R*_Li_ and *R*_Mg_ are their respective rejections. *SF*_Li/Mg_ interprets selectivity as the ratio between the abundance of Li^+^ relative to Mg^2+^ in the permeate compared to their abundance in the feed. Despite its wide acceptability, *SF*_Li/Mg_ has significant limitations in assessing membrane effectiveness for Li^+^ extraction. For instance, according to Equation 3, *SF*_Li/Mg_ becomes highly sensitive to *R*_Mg_, especially when *R*_Mg_ approaches 100%, a very common scenario for high-performance NF membrane.^36,37,55,179,192^ Specifically, the denominator (1-*R*_Mg_) approaches zero when *R*_Mg_ closes to 100%, leading to an extremely high *SF*_Li/Mg_, even when Li^+^ is also effectively rejected. In such cases, *SF*_Li/Mg_ can yield misleadingly high values even when Li^+^ permeation is low, failing to reflect the primary objectives of Li^+^ extraction processes: maximizing Li^+^ recovery (e.g., a high percentage of the total Li^+^ in the feed that ends up in the permeate) and purity (e.g., a high concentration of Li^+^ in the permeate) in the permeate. This critical limitation underscores the necessity for developing new performance metrics that more accurately reflect the dual goals of NF-based Li^+^ extraction and provide a more robust foundation for evaluating and optimizing NF membrane performance in the context of Li^+^ extraction from complex brine solutions (more details can be found in Section 3.2 and 3.3).

### 3.2 Correlation of Li^+^ Purity and Recovery

Recent advancements in performance evaluation metrics for NF-based Li^+^ extraction processes have significantly enhanced the ability to assess membrane efficacy. Wang et al. proposed the use of Li^+^ purity and Li^+^ recovery^32^ as more comprehensive and practically relevant indicators, offering improved insights into solute–solute separation for resource recovery using NF membranes. In a simplified dual Li^+^/Mg^2+^ separation system, the permeate Li^+^ purity, η, can be defined as the mass fraction of Li^+^ in the NF permeate stream:^32^
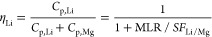
4where MLR is the Mg^2+^-to-Li^+^ ratio in the feed solution. Obviously, improving Li^+^ purity, which aligns well with the goal of improving *SF*_Li/Mg_ in the conventional metrics, is the primary motivation for performing Li^+^/Mg^2+^ separation. That is, NF membranes with high *SF*_Li/Mg_ values tend to produce permeate streams with high Li^+^ purity for a given MLR. High Li^+^ purity is crucial for preventing the precipitation of Li^+^ (e.g., Li_2_CO_3_) with unacceptable levels of impurities (e.g., MgCO_3_), which could greatly undermine the efficiency and sustainability of the Li^+^ extraction process.^193^

Another important metric, Li^+^ recovery (LiR), can be defined as the mass fraction of Li^+^ in the feed that permeates through the membrane, which can be expressed in Equation 5:
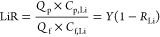
5where *Q*_f_, and *Q*_p_ are volumetric flow rates of the feed and permeate streams and *Y* is water recovery. Notably, this metric challenges the traditional pursuit of high solute rejection, as high Li^+^ passage (i.e., high solute-water selectivity) is beneficial for improving Li^+^ recovery from salt-lake brines.^32^

While the framework correlating Li^+^ purity and Li^+^ recovery provides a more direct and comprehensive assessment for evaluating and optimizing high-quality Li^+^ production, existing studies appear to be limited to coupon-scale analysis.^56^ To address these limitations, we will evaluate the applicability of the state-of-the-art performance evaluation metrics for the NF-based Li^+^ extraction process at a system scale in Section 3.3, aiming to bridge the gap between laboratory-scale performance indicators and the complex realities of industrial-scale Li^+^ extraction, providing a more holistic framework for assessing and optimizing NF membrane technologies in this critical application.

### 3.3 System-Scale Analysis

As previously discussed, a new trade-off correlation between Li^+^ to water selectivity (*B*_Li_*/A*) that relates to Li^+^ recovery and Li^+^ to Mg^2+^ selectivity (*B*_Li_/*B*_Mg_) that relates to Li^+^ purity was recently proposed,^32^ where *B* is the solute permeability coefficient (more details can be found in Supporting Information S2). These factors are considered more important than traditional factors such as water permeance, solute rejection, energy consumption, and etc.,^32^ primarily due to the high economic value of Li^+^. Nevertheless, a rigorous system-level analysis of this framework has yet to be established. In this section, we will employ the finite element method (more details can be found in Supporting Information S2) to investigate the complex interplay of water permeance (*A*), *B*_Li_*/A*, and *B*_Li_/*B*_Mg_ on system-scale membrane performances. Our comprehensive analysis encompasses key performance indicators related to specific energy consumption, system stability, Li^+^ purity, and Li^+^ recovery. This approach aims to optimize NF membranes for Li^+^ extraction at system level, bridging the gap between material-level design principles and practical application.

**Figure 4:**
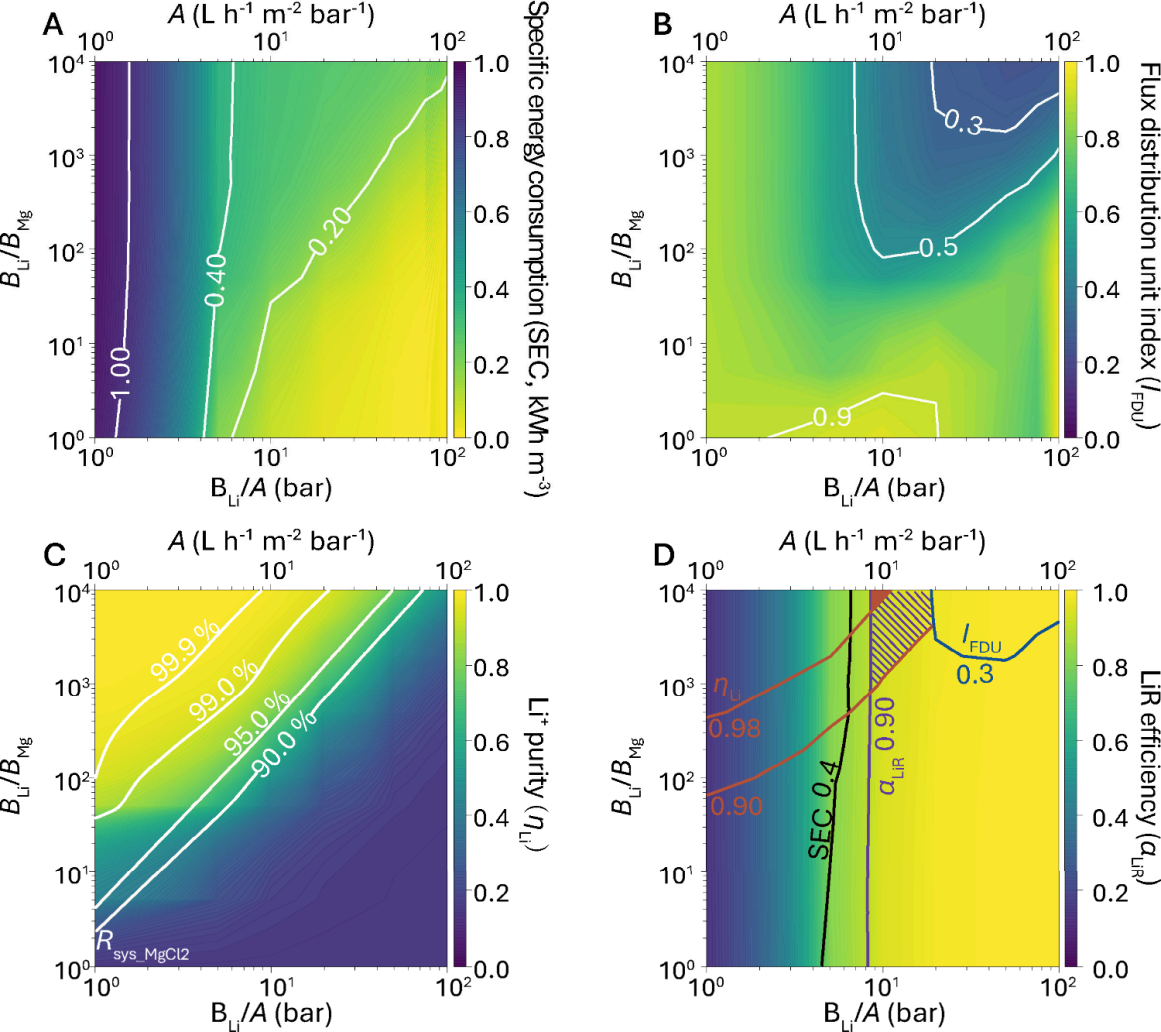
**A system-scale assessment of NF-based LiCl/MgCl**_**2**_**separation in single-pass filtration**. Color contour maps of (A) specific energy consumption (SEC), (B) flux distribution unit index (*I*_FDU_), (C) Li^+^ purity (η_Li_), and (D) LiR efficiency (α_LiR_) for a binary LiCl/MgCl_2_ salt system at water recovery (*Y*) of 80%. To identify the sweet spot, contour lines of SEC of 0.4 kWh·m^–3^; *I*_FDU_ of 0.3, η_Li_ of 0.90 and 0.98, and LiR efficiency of 0.9 are superimposed. The solid area in red represents the region bounded by the lines of SEC = 0.4 kWh·m^–3^, *I*_FDU_ = 0.3, η_Li_ = 0.98, and α_LiR_ = 0.9, whereas the shaded area in purple represents the region bounded by the lines of SEC = 0.4 kWh·m^–3^, *I*_FDU_ = 0.3, η_Li_ = 0.9, and α_LiR_ = 0.9. For all subfigures, the horizontal axis is the Li to water selectivity (*B*_Li_/*A*) or water permeance (*A*), where the numerical value of *B*_Li_/*A* is set to be equal to that of the water permeance *A* (i.e., *B*_Li_ ∝ *A*^2^). This assumption is reasonable considering that solute permeance often increases quadratically with water permeance.^194^ The vertical axis is the Li^+^ to Mg^2+^ selectivity (*B*_Li_/*B*_Mg_). The feedwater contains 3.4 mM LiCl (corresponding to an osmotic pressure of 0.15 bar) and 19.5 mM MgCl_2_ (corresponding to an osmotic pressure of 1.35 bar) with a total osmotic pressure of 1.5 bar, which is representative of salt-lake brines adopted from the ref (32). The average water flux in the system is set to 20 L·m^–2^·h^–1^, a commonly adopted water flux for NF system.^194^ The calculation of SEC is based on our previous work,^56^ where energy recovery device (ERD) is not considered as its use for NF is rare so that the ERD efficiency is set to zero in the analysis.

Figure 4A presents the color contour map illustrating the specific energy consumption (SEC) as a function of membrane separation properties (*A*, *B*_Li_*/A*, *B*_Li_/*B*_Mg_) for LiCl/MgCl_2_ separation. The analysis considers a feed composition containing 3.4 mM LiCl and 19.5 mM MgCl_2_ at a water recovery of 80%. These concentrations correspond to Li^+^ and Mg^2+^ concentrations of ∼25 and ∼500 mg L^–1^, respectively, which are commonly found in Li^+^ - containing salt-lake brines.^32^ In our analysis, we set the numerical value of *B*_Li_*/A* equal to that of *A*, implying *B*_Li_ ∝*A*^2^. This relationship aligns with the literature reporting a quadratic increase in solute permeance with the increased water permeance, indicating a dramatic decline in selectivity as water permeance increases.^194^ In general, increasing water permeance is effective in reducing SEC (Figure 4A). This SEC reduction is more pronounced in the low water permeance range (1–10 L·m^–2^·h^–1^·bar^–1^) and becomes less evident in the high permeance range of >10 L·m^–2^·h^–1^·bar^–1^.^56^ As can be derived from Equation 1, the required hydraulic pressure (Δ*P* = *J*_w_/*A* + Δπ_m_) is governed by two factors: frictional resistance (*J*_w_/*A*) and transmembrane osmotic pressure difference (Δπ_m_). In the low water permeance range, *J*_w_/*A* dominates over Δπ_m_, leading to greatly reduced SEC when increasing water permeance. As a result, the iso-SEC lines are nearly perpendicular to the *x*-axis, indicating SEC’s relative insensitivity to changes in Δπ_m_ resulting from increased membrane selectivity. In contrast, in the high water permeance range, SEC becomes increasingly dependent on membrane selectivity, attributable to the greater influence of Δπ_m_ relative to *J*_w_/*A* across the membrane.

We further analyzed the flux distribution unit index (*I*_FDU_) across the NF system in Figure 4B. According to our previous work,^56^*I*_FDU_ is defined as the ratio of average water flux within the system to the maximum water flux, usually occurring in the lead membrane element. This index, *I*_FDU_, ranging from ∼0 (highly nonuniform flux distribution) to ∼1 (ideally uniform flux distribution), could reflect the nonuniformity of water flux distribution in the system, which could be indicative of potential system failures due to severe concentration polarization and/or aggregated fouling issues. Intriguingly, Figure 4B demonstrates that low *I*_FDU_ values (≤0.3), where the lead element water flux is over three times the average flux of the system, are observed in the upper right quadrant in the plot, which could potentially incur severe CP and/or fouling issues. Indeed, the combination of high water permeance and high selectivity could lead to reduced contribution of frictional resistance (*J*_w_/*A*) and enhanced contribution of the transmembrane osmotic pressure difference (Δπ_m_).^56^ As a result, the flux distribution becomes more sensitive to the increased solute concentration in the retentate along the pressure vessel after partial water recovery.^56^ It is interesting to note that although our analysis reveals the potential risks of flux nonuniformity for the membranes located in the upper right quadrant in Figure 4B, traditional wisdom often believes that the combinations of high water permeance (*A*), high Li^+^ to water selectivity (*B*_Li_/*A*), and high Li^+^ to Mg^2+^ selectivity (*B*_Li_/*B*_Mg_) are often considered as the “ideal performance”.^162,195^ Therefore, caution should be exercised when interpreting this “ideal” region in future analyses.

**Figure 5:**
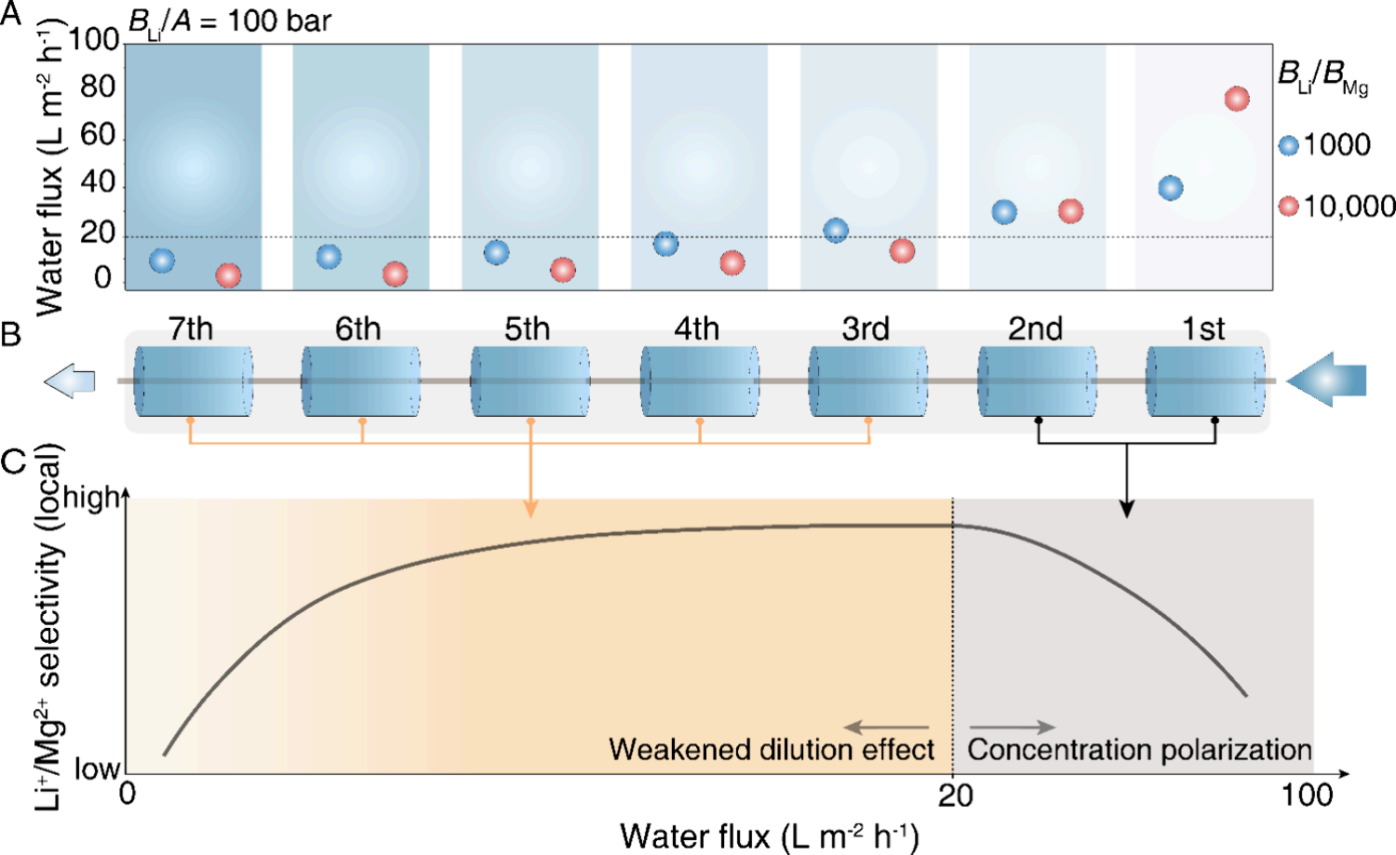
**Flux distribution within a 7-element pressure vessel and a semiquantitative correlation between localized water flux and localized Li**^**+**^**/Mg**^**2+**^**selectivity for a binary LiCl/MgCl**_**2**_**salt system at a water recovery (*****Y*****) of 80%**. (A) In our analysis, the water permeance *A* and Li to water selectivity *B*_LiCl_/*A* are fixed at 100 L·m^–2^·h^–1^·bar^–1^ and 100 bar, respectively, with two different sets of LiCl/MgCl_2_ selectivity (*B*_Li_/*B*_Mg_) of 1000 and 10,000. The system average water flux of 20 L·m^–2^·h^–1^ is superimposed in plot (A) in black dashed line, (B) The schematic illustration of a 7-element pressure vessel with flow direction from right (feed stream) to left (permeate stream), (C) A semiquantitative correlation between localized water flux and localized Li^+^/Mg^2+^ selectivity, suggesting an impaired Li^+^/Mg^2+^ selectivity due to the enhanced concentration polarization effect at the high flux zone (i.e., significantly above the system average flux) in the first and second element and the weakened dilution effect at the low flux range (i.e., significantly below the system average flux) in the last few tail elements. Plot (C) of the figure was adapted with permission from ref (32) (Copyright 2023 Springer Nature).

To gain a more in-depth understanding, Figure 5 elucidates how system flux nonuniformity, caused by highly permeable and highly selective membranes, affects system selectivity by plotting the localized water flux against the localized selectivity. As the system water flux becomes progressively nonuniform (indicated by a low *I*_FDU_ value) incurred by the high-performance membranes, the lead element experiences disproportionately high water flux (≫ the average water flux of 20 L·m^–2^·h^–1^). This localized high flux (e.g., *J*_w_ ∼ or > *k*, Equation S3) can induce severe CP phenomenon, significantly impairing the localized Li^+^/Mg^2+^ selectivity (thus affecting Li^+^ purity based on eq 4). Moreover, the disproportionate water flux in the lead element has cascading effects on the remaining elements. For the last few tail elements, the redistributed permeate water results in much lower localized water flux compared to the average system water flux. According to the literature,^32^ low water flux is also detrimental to the localized Li^+^/Mg^2+^ selectivity due to the substantially reduced rejections of all solutes, a consequence of the weakened dilution effect (i.e., similar salt flux *J*_s_ with reduced water flux *J*_w_, resulting in a higher solute concentration in the permeate [= *J*_s_/*J*_w_]). Indeed, as *SF*_Li/Mg_ is more sensitive to *R*_Mg_ than *R*_Li_, this across-the-board reduction in solute rejection could cause a dramatic drop of *SF*_Li/Mg_ values. Therefore, the combined effects of enhanced CP and the weakened dilution effect incurred by the low *I*_FDU_ collectively contribute to the impaired system Li^+^/Mg^2+^ selectivity. These effects are further exacerbated by adopting membrane with higher selectivity (Li^+^/Mg^2+^ selectivity of 10,000 versus 1000 shown in Figure 5A). Therefore, we call for researchers and practitioners to adopt a more holistic approach when designing and evaluating “high-performance” NF membranes for lithium extraction, considering not only separation performance at coupon scale but also additional factors at system scale (e.g., flux distribution uniformity, local water flux, and local selectivity).

System-scale Li^+^ purity (η_Li_), defined as the cumulative mass fraction of Li^+^ product in the final NF permeate stream, is another important parameter to assess the success of Li^+^/Mg^2+^ separation.^162,195^ While the industrial requirements of Li^+^ product purity typically range from ∼98.0 to 99.9%,^162^ post-treatments, such as additional chemical purification and precipitation, could further increase Li^+^ purity from 90.0 to 95.0% to ≳98.0%.^162,193^Figure 4C presents the color contours for the Li^+^ purity in the product water for different combinations of water permeance, LiCl to water selectivity, and LiCl/MgCl_2_ selectivity at the water recovery of 80%. In general, excellent Li^+^ purity (>90%), i.e., high Li^+^ concentration in the product water as represented by yellow color, is achieved over the upper left region with high LiCl/MgCl_2_ selectivity and moderate water permeance. Our system-scale findings reveal a stark contrast compared to the conventional evaluation framework,^32,195^ where the upper right quadrant in the diagram with simultaneously high *A*, *B*_Li_*/A*, and *B*_Li_/*B*_Mg_ values is often regarded as the “ideal” spots. For instance, an “ideal” NF membrane at a given membrane separation properties (e.g., *A* = 100 L·m^–2^·h^–1^·bar^–1^, *B*_Li_*/A* = 100 bar, and *B*_Li_/*B*_Mg_ = 10,000) could only result in a system-scale Li^+^ purity as low as ∼50.0% in the single-pass filtration. In contrast, a conventionally less optimal membrane (e.g., *A* = 10 L·m^–2^·h^–1^·bar^–1^, *B*_Li_*/A* = 10 bar, and *B*_Li_/*B*_Mg_ = 1000) could achieve a η_Li_ value of ∼90%. To gain more mechanistic insights, system rejection lines for MgCl_2_ (*R*_sys_MgCl2_) of 99.9%, 99%, 95%, and 90% are superimposed in the same plot. While Li^+^ purity of >98%, meeting the typical industrial requirements, is generally obtained for *R*_sys_MgCl2_ > 99.9%, a less stringent requirement for Li^+^ purity of >90% (which needs additional chemical purification/precipitation before industrial use) could lessen the *R*_sys_MgCl2_ requirement to ∼95–99%. In this regard, our results suggest that NF membranes with moderate water permeance (∼10 L·m^–2^·h^–1^·bar^–1^), high *B*_Li_/*B*_Mg_ value of ∼ or >1000, and high *R*_sys_MgCl2_ of ∼ or >95% are required for achieving the satisfactory Li^+^ purity of ≳90% in the single-pass filtration. Indeed, as membrane water permeance increases, a more dramatic increase in solute permeance could occur.^56^ Together with the negative impact of the low *I*_FDU_ as we have illustrated in Figure 5, these combined effects could substantially reduce the rejection rates of all solutes, causing a dramatic drop of *SF*_Li/Mg_ value and thus impairing system Li^+^ purity. Future studies could explore other process designs, such as extending the current single-pass to multipass configuration to further improve Li^+^ purity.

In addition to the preceding metrics, we further explore the impact of membrane separation properties on Li^+^ recovery,^32^ which is defined as the mass fraction of Li^+^ from the feed stream that eventually ends up in the permeate stream. To ensure consistency in our analysis, we introduce a new recovery indicator—Li^+^ recovery efficiency (α_LiR_), which can be obtained by normalizing Li^+^ recovery to water recovery as shown in Equation (S12). It is worthwhile to note that this LiR efficiency (α_LiR_) is identical to the system-level Li^+^ passage. In general, a higher α_LiR_ value is preferred to ensure the high recovery of Li^+^ from the feed stream. Figure 4D presents the color map of Li^+^ recovery efficiency, where a higher *B*_Li_*/A* value tends to enhance Li^+^ recovery, underpinning the importance of *B*_Li_*/A* in Li^+^ extraction process, despite that this metric has been far less discussed previously compared to water permeance and LiCl/MgCl_2_ selectivity. To establish a more systematic framework in a typical Li^+^ extraction process, we further superimpose the SEC (black colored line), flux distribution uniformity index (blue colored line), and Li^+^ purity (red colored line) in Figure 4D, which allows us to identify an operational window for a sweet spot (shaded area in purple) to fulfill the targets of moderate energy consumption (<0.4 kWh·m^–3^), acceptable system stability (*I*_FDU_ > 0.3), moderate Li^+^ purity (η_Li_ > 0.9), and high Li^+^ recovery (α_LiR_ > 0.9) simultaneously. Following these criteria, the highly permeable and highly Li^+^-selective NF membranes based on the traditional definition of ideal membranes that are located at the upper-right quadrant of the plot are no longer capable of achieving this target. Alternatively, the combination of moderate water permeance (∼10 L·m^–2^·h^–1^·bar^–1^), moderate Li^+^ to water selectivity (∼10 bar), and high LiCl/MgCl_2_ selectivity (>100) is preferred. For more stringent criteria with the SEC of <0.4 kWh·m^–3^, η_Li_ > 0.98 (highly purified Li^+^ product), acceptable system stability (*I*_FDU_ > 0.3), and α_LiR_ > 0.9, this operational window would greatly shrink (solid area in red) and a more selective NF membrane with high Li^+^ to Mg^2+^ selectivity appears to be more preferred over that of high water permeance or Li^+^ to water selectivity. Indeed, we have demonstrated that membranes with high water permeance together with high selectivity could result in the severe coupled CP and weakened dilution effect (Figure 5C), both of which could greatly impair the system selectivity. We note that the further simultaneous improvement of Li^+^ purity and recovery, and reduction on energy consumption in a single-pass NF process is highly challenging due to the trade-off between Li^+^ recovery and Li^+^ purity at the system level. Future studies should expand membrane research beyond materials and perform more rigorous process analysis and system optimization to exceed the trade-off limits (e.g., multipass design).

## 4 Concluding Remarks and Future Perspectives

**Figure 6:**
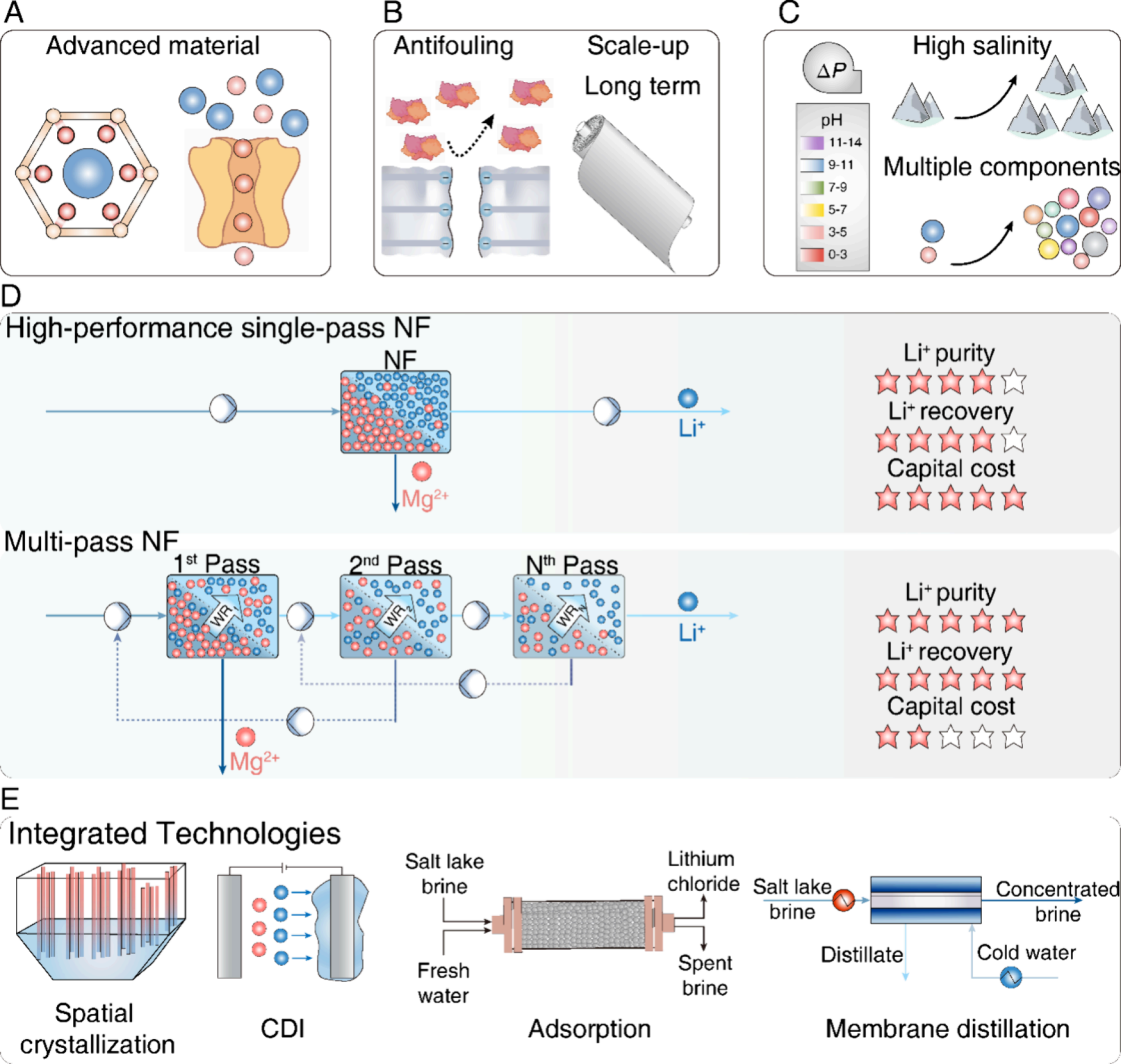
**Schematic illustrations for designing and optimizing highly Li**^**+**^**-selective NF or integrated technologies for real-world Li**^**+**^**extraction. (A) Tailoring membrane materials for enhanced Li**^**+**^**selectivity**. Advanced NF membrane materials incorporating coordination chemistry (left), such as crown ethers or metal–organic frameworks with tailored sizes and chemical functionalities, and/or leveraging biomimetic nanochannels (right) inspired by aquaporin or ion-specific transport pathways, to facilitate selective Li^+^ passage. **(B) Prioritizing performance metrics for industrial feasibility**. Evaluation of membrane performance should focus on antifouling properties, scalability potential, and long-term stability. **(C) Utilizing realistic brine conditions for accurate assessment**. Filtration experiments using feed solutions should closely mimic complex salt-lake brine compositions, with investigations into the influence of applied pressure, pH, and temperature on Li^+^ transport. **(D) Optimizing NF system design and the ratings**. Comparison of high-performance single-pass and multipass NF systems, with corresponding ratings for Li^+^ purity, recovery, and capital cost. **(E) Integration with complementary techniques**. Potential synergies between NF and other separation technologies include: (1) Spatial crystallization (leveraging controlled supersaturation for selective Li^+^ crystallization, enhancing overall recovery and purity), (2) CDI (electrochemical method for treating dilute streams or polishing NF permeate, potentially achieving higher overall Li^+^ recovery), (3) Adsorption (integration of selective Li^+^ adsorbents for pre- and/or postpurification), (4) Membrane distillation (thermal-driven process to concentrate NF permeate, moving toward zero liquid discharge and improving overall water recovery). These integrated approaches aim to overcome the limitations of individual technologies, potentially leading to more efficient, sustainable, and economically viable Li^+^ extraction processes.

This review provides a comprehensive summary of the state-of-the-art NF technology for Li^+^ extraction, including advances in modification strategies of NF membranes, identification of key parameters that dictate membrane selectivity, and the re-evaluation of separation performance metrics at system scale. To further advance this field, future work should explore opportunities for advanced membrane materials, process optimization and integration, sustainability, and overcoming challenges related to industrial-scale implementation (Figure 6).

### 4.1 Advanced Membrane Materials

As discussed in Section 2.2, advanced NF membrane targeted for efficient Li^+^ extraction is rapidly evolving toward molecular-level design. While enhancing the membrane’s ability to discriminate between Li^+^ and interfering ions (e.g., Mg^2+^) remains crucial (Figure 6A), current research has been increasingly focused on the synergistic optimization of multiple transport phenomena. Precise engineering of membrane pore size distributions, targeting the narrow range between hydrated Li^+^ ions and Mg^2+^ ions, has been complemented by the manipulation of electrostatic interactions and solvation dynamics at the membrane-solution interface.^37,41^ For instance, the incorporation of novel nanomaterials (e.g., MXenes^196^) into the existing membrane rejection layer could manipulate the pore size in the rejection layer with tailored Li^+^ transport properties. Furthermore, biomimetic approaches, such as porous organic channels inspired by natural ion selectivity, could yield synthetic structures with Li^+^-specific affinity, promisingly improving Li^+^ extraction.^20,26,110,197^

Despite the promising advanced materials, the simultaneous enhancement of water permeance and Li^+^ recovery/selectivity presents a dilemma. Future research may explore high free-volume polymers and novel membrane nanoarchitectures to improve water permeance and Li^+^ recovery/selectivity simultaneously.^46,54,198^ Notable examples include the creation of vertically aligned nanochannels that minimize tortuosity and the incorporation of zwitterionic polymer brushes that can modulate local hydration environments.^199,200^ Stimuli-responsive membranes capable of dynamically modulating properties in response to brine composition fluctuations present a frontier in adaptive separation technology.^20,120,201^ These smart membranes could potentially self-optimize their performance in real time, adapting to variations in feed composition and process conditions. The exploration of organic–inorganic hybrid materials is evolving toward the creation of membranes with hierarchical structures that combine the stability of inorganic components with the selectivity of organic moieties (Figure 6B). Future validation protocols for these advanced membrane nanoarchitectures should move beyond simple separation performance tests. Comprehensive characterization techniques, including *in situ* spectroscopic methods and advanced imaging technologies, could be more important for understanding the dynamic behavior of these membranes under realistic operating conditions (Figure 6C, see compositions of exemplified worldwide salt-lake brines in Supporting Information Table. S2). This necessitates establishing standardized protocols using multicomponent synthetic brines and conducting long-term performance studies with real salt-lake brines.^7,53,65^ One potential strategy is to develop the data-driven approach (e.g., machine learning algorithms) to predict long-term membrane performance based on short-term test data, which could significantly accelerate the optimization process.

### 4.2 Process Optimization and System Integration

Existing NF studies, typically performed at bench-scale at nearly zero water recovery, lack the practical demonstration of highly efficient lithium extraction.^32,36,107,174^ Future research should adopt system-level approaches, addressing the multidimensional optimization that balances Li^+^/Mg^2+^ separation factor, Li^+^ recovery, water permeance, and energy consumption.^56^ This necessitates the development of advanced process modeling tools that can accurately predict membrane performance under varying operational conditions (e.g., the effects of concentration polarization and scaling in high-salinity environments). It is also worthwhile to note that recent fluctuation in Li^+^ prices has shifted the focus toward operational costs, particularly energy consumption.^202^ This economic shift necessitates a re-evaluation of the balance between membrane performance and energy efficiency at the system level. For instance, low permeance membranes, while potentially offering higher Li^+^ selectivity, now may face limitations due to their higher energy consumption (see Figure 4A). When using low permeance membranes, higher pressures are required to maintain the desired water flux, leading to significantly increased energy consumption.^203^ This increased energy costs can significantly impact operational costs, potentially offsetting the benefits of the economic values of Li^+^ product. Future studies should perform a wholistic approach to balance the economic value of Li^+^ product and energy consumption during the extraction process.

The concept of a single-pass, high-performance NF operation (Figure 6D) capable of achieving moderate Li^+^ purity and recovery represents a promising research direction. Nevertheless, the inherent trade-off between Li^+^ purity and recovery in single-pass processes, as demonstrated in Figure 4D, underscores the need for novel process configurations. Multipass NF systems with brine recirculation offer a potential solution (see Figure 6D), demonstrating remarkable system *SF*_Li/Mg_ (e.g., >4500) and Li^+^ recovery rates (e.g., >95%).^162^ Nevertheless, since capital costs and process complexity of the multipass process significantly increase, a rigorous techno-economic assessment (TEA) is therefore required to systematically compare the economic benefits. Novel membrane modules and spacer designs are other promising avenues that could achieve optimal hydrodynamic conditions during the Li^+^ extraction process, which could potentially mitigate membrane fouling and CP issues. However, the optimization of these systems must go beyond simply maximizing separation performance. Future research should focus on developing intelligent control systems that can dynamically adjust operational parameters to maintain optimal performance under fluctuating feed conditions.

The complex composition of salt-lake brines and the challenges in simultaneously achieving high Li^+^ purity and recovery necessitate the integration of NF with other DLE techniques to overcome individual technological limitations (Figure 6E). Promising examples include combining NF with spatially separated crystallization, which has shown remarkable selectivity by manipulating metastable zone widths of different salts,^10^ potentially allowing for a two-stage process of initial NF purification followed by fine-tuned crystallization. Coupling NF with capacitive deionization (CDI) could exploit the selectivity of CDI for streams containing low Li^+^ concentrations, particularly in treating NF permeate for polishing steps.^204,205^ The combination of NF with electrochemical extraction methods presents the potential to enhance overall process efficiency by merging NF’s high throughput with the high selectivity of electrochemical techniques.^16,25^ Additionally, integrating NF with membrane distillation could potentially address high-salinity retentate handling, moving toward zero liquid discharge operations and improving overall water recovery.^30,206,207^ Another promising integration is adsorption, using selective Li+ adsorbents for pre- and/or postpurification to improve the Li^+^ extraction efficiency.^30^ As these integrated systems evolve, advanced process simulation tools incorporating dynamic modeling and machine learning algorithms will be crucial for optimizing multitechnology configurations. Comprehensive techno-economic and life cycle assessments will be essential to demonstrate the viability and sustainability of these integrated approaches compared to conventional methods.^7,12^ These assessments need to consider not only operational costs and environmental impacts but also process flexibility, robustness to feed variability, and potential for heat and mass integration across different process units. The synergistic combination of multiple DLE techniques in future Li^+^ extraction processes holds the promise of achieving excellent treatment efficiency, sustainability, and economic viability. However, realizing this potential will require overcoming challenges in process complexity, control system design, and scale-up issues. Future research should focus on developing modular, adaptable process designs tailored to specific brine compositions and local environmental conditions.

### 4.3 Sustainability and Environmental Considerations

**Figure 7:**
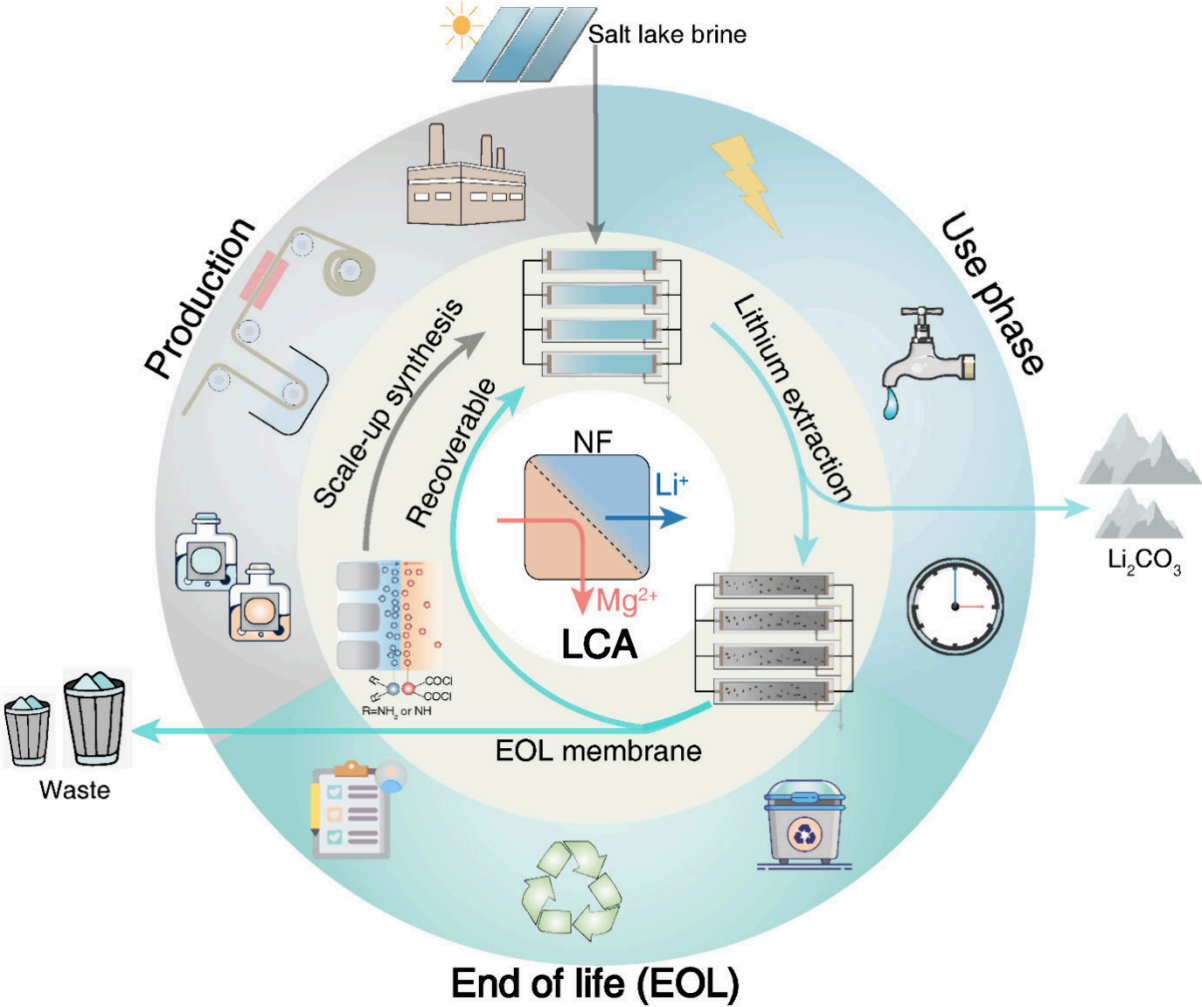
**Comprehensive life cycle assessment (LCA) for sustainability evaluation of NF-based Li**^**+**^**extraction**. In the production phase, factors should be associated with raw material acquisition, membrane fabrication, chemical waste generation, scale, and module assembly. For the use phase (e.g., Li^+^ extraction), energy consumption, water usage, reagent inputs, separation efficiency, Li^+^ purity, recovery rate, and membrane lifetime should be assessed. For end-of-life (EOL) management, researchers should consider membrane disposal options, recycling potential, environmental footprint, opportunities for circular economy implementation, and repurposing strategies. This comprehensive approach ensures that sustainability is evaluated at every stage of the membrane’s lifecycle, from initial production to final disposal or recycling, promoting more environmentally responsible and economically viable NF-based Li^+^ extraction processes. By considering this wide range of factors, researchers and practitioners can make informed decisions to optimize the overall sustainability of NF-based Li^+^ extraction technologies, balancing environmental protection with economic feasibility and resource conservation.

As NF-based Li^+^ extraction technologies advance toward large-scale implementation, their environmental implications are poised to reshape the sustainability landscape of the global Li^+^ supply chain. While current life cycle assessment (LCA) methodolgies^7,208^ provide a foundation for environmental impact evaluation on conventional Li^+^ extraction methods, such as evaporation ponds and hard rock mining,^208−210^ LCA studies on NF-based processes for Li^+^ extraction remains notably scarce.^211^ Given the unique challenges posed by the characteristics of salt-lake brines, the scope of LCA should consider technological novelty, product value, circular economy potential, ecosystem assessment, and disposal and regeneration (Figure 7).^17,212,213^ To more accurately reflect the environmental impact of the NF-based process in the salt-lake region, future LCAs should address key areas, including membrane life cycle analysis in hypersaline environments, brine-specific impact categories, energy efficiency evaluation, Li^+^ recovery rate and purity, and integration with complementary technologies such as previously mentioned CDI (Figure 6E). Scalability impacts, circular economic considerations, and regional variability of salt-lake ecosystems should also be considered. In this regard, dynamic modeling approaches will be crucial to account for rapid advancements in NF membrane design and process optimization.^7^ Rigorous comparative analysis between NF-based separation processes and conventional and other emerging extraction methods, considering water consumption, carbon footprint, land use, and Li^+^ recovery rate and purity, will inform policy and investment decisions. In addition, prospective or ex-ante LCAs will become increasingly important during the early stages of NF technology development and deployment.^209,214^ These forward-looking assessments will guide design decisions and help mitigate potential environmental impacts proactively, ensuring that sustainability considerations are integrated from the outset of technology development. Finally, integrating tech-economic analysis (TEA) into LCA would be important for weighing the environmental implications and the economic viability of the NF-based lithium extraction processes.^4^

By unleashing these opportunities and addressing these challenges, researchers or practitioners could pave the way for developing high-performance NF membranes with optimized systems capable of meeting the escalating global lithium demand in a more efficient and sustainable manner. Although the context of this review focuses on Li^+^ extraction, the evaluation framework and technical approaches can be readily extended to evaluate other solute–solute separations to achieve efficient resource recovery and recycling. This holistic approach developed in this review is expected to guide future endeavors in advanced membrane designs, process innovations, and economic optimizations for extracting target solutes.

## Supporting Information Available

S1. Explainable machine learning methods; S2. System-level simulation; S3. Summary of literature data of NF membrane for Li^+^ extraction; S4. Summary of typical salt-lake brines composition (PDF)
