# Supplementary material for: Nanofiltration Membranes for Efficient Lithium Extraction from Salt-Lake Brine: A Critical Review
Source: ACS Environ Au. 2024 Nov 20;5(1):12–34. doi: 10.1021/acsenvironau.4c00061 (PMC11740921; doi:10.1021/acsenvironau.4c00061)
Supplement: Supplementary file 1 — vg4c00061_si_001.pdf [file vg4c00061_si_001.pdf]

Supporting Information

Nanofiltration Membranes for Efficient Lithium Extraction from Salt-lake Brine: A Critical Review

Ming Yong,[†, ‡, §] Yang Yang,[∥] Liangliang Sun,[‡, §] Meng Tang,[‡, §] Zhuyuan Wang,[†] Chao Xing,[†] Jingwei Hou,[⊥] Min Zheng[#], Ting Fong May Chui,[∥] Zhikao Li[*, ‡, §], and Zhe Yang[*,†]

[†]Dow Centre for Sustainable Engineering Innovation, School of Chemical Engineering, The University of Queensland, Brisbane, QLD 4072, Australia

[‡]Department of Chemical and Biological Engineering, Monash University, Clayton, VIC 3800, Australia

[§]Suzhou Industrial Park Monash Research Institute of Science and Technology, Suzhou, 215000, Jiangsu Province, China

[∥]Department of Civil Engineering, The University of Hong Kong, Pokfulam, Hong Kong, 999077, SAR China

[⊥]School of Chemical Engineering, The University of Queensland, St Lucia, QLD 4072 Australia

[#]Water Research Centre, School of Civil and Environmental Engineering, University of New South Wales, Sydney, New South Wales, Australia

[*]To whom all correspondence should be addressed.

Tel: +86 13771807493, E-mail address: zhikao.li@monash.edu

Tel: + 61 7334 66464, E-mail address: zhe.yang@uq.edu.au

24 pages with two tables.

Contents

S1. Explainable machine learning methods S3

S1.1. Training machine learning models S3

S1.2. SHAP method S4

S2. System-level simulation S5

S3. Summary of literature data for lithium extraction S8

S4. Summary of typical salt-lake brines composition S17

References S19

S1. Explainable Machine Learning Methods

S1.1 Training Machine Learning Models

In this study, machine learning models were developed using the gradient-boosted trees algorithm.[1] We utilized the XGBoost library in R due to its advanced regularization methods, computational efficiency, and proven performance in various machine learning tasks.[2,3]

To assess the predictive performance of the machine learning algorithm, we employed a nested cross-validation method. The inner loop optimizes the hyperparameters, while the outer loop evaluates the generalization performance. Both loops use five folds. Each outer loop iteration can be considered an experiment assessing the model's predictive performance.

XGBoost uses several hyperparameters to regulate the training process and to specify the model structure, including step size shrinkage, maximum tree depth, and subsample ratio of the training instances. During the inner loop, these hyperparameters were optimized using a Bayesian optimization method via the mlrMBO[4] library in R. The optimization problem aimed to minimize prediction errors on the validation sets, with hyperparameters as the decision variables. Upon completing the optimization, an XGBoost model was trained on the combined training and validation datasets and evaluated on the test dataset in the outer loop.

S1.2 SHAP Method

In this study, the SHapley Additive exPlanations (SHAP) method was used to quantify the contribution of each feature to the predictions made by the XGBoost model for specific input samples[5]. A SHAP value quantifies the marginal contribution of a feature to the predicted value. SHAP values obey several important axioms, such as the sum of the SHAP values for all features equals the difference between the predicted value and the expected value of the model output. We used functions from the XGBoost and SHAPforxgboost[6] R libraries to compute the SHAP values. The SHAP values of different features of each input and the mean absolute SHAP value of each feature across all the samples were calculated to explain the contribution of different factors to the membrane's performance.

S2. System-level Simulation

The solute flux (J_s) through a dense membrane is described as follows:[7]

$$J_s = B\Delta C_m \tag{S1}$$

where B is solute permeance or solute permeability coefficient. ΔC_m is the concentration difference across the membrane. According to the film theory,[7] ΔC_m is related to the corresponding bulk solution value (C_b) and that in the permeate water (C_p):

$$\Delta C_m = f_{cp}(C_b - C_p) \tag{S2}$$

$$f_{cp} = \exp(\frac{J_w}{k}) \tag{S3}$$

where the factor f_{cp} describes the effect of concentration polarization (CP) and is calculated using J_w and the overall mass transfer coefficient k is calculated based on our previous study.[7]

To perform the system-scale analysis, we further adopt a one-dimensional finite element model with the feed water containing both $MgCl_2$ and LiCl. Mass balance equations of water, $MgCl_2$ and LiCl are given by:

$$Q_f = Q_c + Q_p \tag{S4}$$

$$Q_f C_{f\_MgCl_2} = Q_c C_{c\_MgCl_2} + Q_p C_{p\_MgCl_2} \tag{S5}$$

$$Q_f C_{f\_LiCl} = Q_c C_{c\_LiCl} + Q_p C_{p\_LiCl} \tag{S6}$$

Eq. S4-S6 can be written in their respective differential forms:

$$\mathrm{d}Q_c = -J_w \mathrm{d}A_m = -J_w nW\mathrm{d}L \tag{S7}$$

$$\mathrm{d}(Q_c C_{c\_MgCl_2}) = -J_{s\_MgCl_2} \mathrm{d}A_m = -J_{s\_MgCl_2} nW\mathrm{d}L \tag{S8}$$

$$\mathrm{d}(Q_c C_{c\_LiCl}) = -J_{s\_LiCl} \mathrm{d}A_m = -J_{s\_LiCl} nW\mathrm{d}L \tag{S9}$$

where dA_m is the membrane surface area over a differential length dL of a membrane module, n and W are the number of membrane leafs and the effective leaf width of a given membrane module, respectively. More detailed module information can be found from our previous study.[7] By discretizing each membrane module into a number of segments, eq S7-S9 (mass balance equations) can be coupled with equations for fluxes of $MgCl_2$ and LiCl to solve for the localized fluxes J_w, J_{s_MgCl2}, and J_{s_LiCl}. The localized properties of the concentrate (flow rate, $MgCl_2$ concentration, and LiCl concentration) at this segment are then updated and adopted as the inputs for the next segment. Such numerical iterations are realized using the Matlabs software (Mathworks, USA).[7]

The finite element method presented in this section allows for the evaluation of the required SEC and a variety of system-level performance metrics, including system rejection, localized water flux, etc. For a binary salt system, both R_{sys_MgCl2} and R_{sys_LiCl} can be obtained. Accordingly, one can evaluate the system-scale separation factor $SF_{sys\_Li/Mg}$ by:

$$SF_{sys\_Li/Mg} = \frac{(C_{LiCl} / C_{MgCl_2})_p}{(C_{LiCl} / C_{MgCl_2})_f} = \frac{1 - R_{sys\_LiCl}}{1 - R_{sys\_MgCl_2}} \tag{S10}$$

To assess the success of the system-scale Li^+/Mg^{2+} separation, the system-scale Li^+ purity, denoted as η_{sys_Li}, is defined as the cumulative mass fraction of Li^+ in the NF permeate stream at the system level:

$$\eta_{\mathrm{sys\_Li}} = \frac{C_{\mathrm{sys\_Li,p}}}{C_{\mathrm{sys\_Li,p}} + C_{\mathrm{sys\_Mg,p}}} \tag{S11}$$

We can further define a term LiR efficiency α_{LiR} by normalizing lithium recovery with that of water recovery,

$$\alpha_{\mathrm{LiR}} = \frac{Q_{\mathrm{p}} \times C_{\mathrm{p,Li}}}{Q_{\mathrm{f}} \times C_{\mathrm{f,Li}}} / \frac{Q_{\mathrm{p}}}{Q_{\mathrm{f}}} = \frac{C_{\mathrm{p,Li}}}{C_{\mathrm{f,Li}}} = 1 - R_{\mathrm{sys\_LiCl}} \tag{S12}$$

It is worthwhile to note that LiR efficiency α_{LiR} is identical to the system-level Li^+ passage.

S3. Summary of Literature Data of Nanofiltration Membrane for Lithium Extraction.

Table S1. Summary of literature data of nanofiltration membrane for lithium extraction.

Membranes	Charge	MWCO (Da)	Pore size (Å)	Pressure (Bar)	Salinity (mg·L^{-1})	Mg^{2+}/Li^{+} ratio	Water flux $L·m^{-2}·h^{-1}$	Water Permeance. $L·m^{-2}·h^{-1}·bar^{-1}$	$SF_{Li/Mg}$	Ref.
PEI-EDA/TMC/PES	Positive	560	5.4	4	2000	60	21.68	5.42	80.62	8
						40	20.8	5.2	69.2	
						120	20	5	46.79	
PIP/TMC/PSF	Negative	378	3.5	6	2000	27.3	NR*	NR	4.32	9
PIP/TMC/LP8&PSF		275	2.31			27.3	NR	NR	42.1	
PIP/TMC/CP8&PSF		254	2.15			27.3	49.2	8.2	75.86	
PIP/TMC/PES	Negative	212	1.6	6	2100	20	NR	NR	5	10
PIP/TMC/PES-10% IL	Positive	276	4.4			20	NR	NR	68	
PIP/TMC/PES-15% IL	Positive	441	5.6	6	2100	20	NR	NR	19	
PIP/TMC/PES-20% IL	Positive	669	6.9			20	NR	NR	9	
PIP/TMC/PES-40% IL	Positive	550	6.3			20	NR	NR	12	
PIP/TMC/PES-60% IL	Positive	438	5.7			20	NR	NR	13	
PIP/TMC/PSF	Negative	381.3	2.14	10	3000	7.8	379	37.9	15.84	11
				10	5500	15.6	379	37.9	10.44	
				10	10500	31.2	379	37.9	17.89	
				15	10500	31.2	NR	NR	14.62	
PIP/TMC/DA-G4D& PSF	Negative	348.4	1.83	10	3000	7.8	264.3	26.43	44.72	
	Negative			10	5500	15.6	264.3	26.43	94.91	

	Negative			10	10500	31.2	264.3	26.43	60.98	
	Negative			15	10500	31.2	NR	NR	40.41	
PIP/TMC/BA-G4D&PSF	Negative	342.8	1.89	10	3000	7.8	232.9	23.29	97.13	
				10	5500	15.6	232.9	23.29	94.16	
				10	10500	31.2	232.9	23.29	116.8	
				15	10500	31.2	NR	NR	95.28	
PIP/TMC/p-HC-G4D&PSF	Negative	345.1	1.89	10	3000	7.8	267.5	26.75	64.29	
				10	5500	15.6	267.5	26.75	68.77	
				10	10500	31.2	267.5	26.75	72.19	
				15	10500	31.2	NR	NR	36.59	
PEI/TMC/PSF	Positive	399.8	6.4	NR	NR	NR	NR	NR	NR	12
PEI-Cyclen/TMC/PSF	Positive	725.1	6.8	5	2000	5	70	14	6.9	
						10	70	14	7.5	
						20	70	14	8	
						50	70	14	7.4	
1%-PAA/TMC/PK	Positive	232	NR	5	2000	20	28.9	5.78	16.26	13
0.5%-PAA/TMC/PK		246				20	NR	NR	NR	
0.3%-PAA/TMC/PK		276				20	36.95	7.39	82.8	
0.2%-PAA/TMC/PK		315				20	38.35	7.67	31.63	
0.1%-PAA/TMC/PK		302				20	47.8	9.56	12.35	
HPEI-C-PBI	Positive	288	NR	6	500	10	12	2	13.76	14
					1000	10	10	1.67	14.81	
					2000	10	8	1.33	12.72	
DTES&PEI/TMC/PSF	Positive	380	3.8	8	2100	20	34.6	4.33	7.28	15
DTES&PEI/TMC/PSF	Positive	439	3.9			20	42.5	5.31	9.21	

DTES&PEI/TMC/PSF	Positive	553	4.1			20	49.58	6.2	12.95	
DTES&PEI/TMC/PSF	Positive	830	4.6			20	42	5.25	11.35	
DTES/TMC/PSF	Positive	1036	5.6			20	25.7	3.21	9.29	
DTES&PEI/TMC/PSF	Positive	1100	6			20	8.35	1.04	5.91	
PEI/TMC/PSF	Positive	178	NR			20	1.5	0.15	3.05	
PEI/TMC/TA-PSF	Positive	180	2.9	10	2000	20	17	1.7	22.22	16
Cu-PEI/TMC/TA-PSF	Positive	280	3.1			20	29	2.9	26.5	
PEI/TMC/CA&PEI interlayer/PSF	Negative	242	4.41	NR	NR	NR	NR	NR	NR	
PEI/TMC/CA&DSD interlayer/PSF	Negative	253	4.51	NR	NR	NR	NR	NR	NR	
PEI/TMC/HQ&PEI interlayer/PSF	Negative	244	4.41	NR	NR	NR	NR	NR	NR	
PEI/TMC/HQ&DSD interlayer/PSF	Negative	256	4.46	NR	NR	NR	NR	NR	NR	
PEI/TMC/PG&PEI interlayer/PSF	Negative	248	4.48	NR	NR	NR	NR	NR	NR	
PEI/TMC/PG&DSD interlayer/PSF	Negative	252	4.51	NR	NR	NR	NR	NR	NR	
PEI/TMC/Noria&PEI interlayer/PSF	Negative	239	4.35	NR	NR	NR	NR	NR	NR	
PEI/TMC/Noria&DSD interlayer/PSF	Negative	270	4.69	NR	NR	NR	NR	NR	NR	
PEI/TMC/Noria-PG&PEI interlayer/PSF	Negative	238	4.34	10	2000	20	NR	NR	88.6	17
					4000	20	NR	NR	58.7	
					6000	20	NR	NR	37.9	
					2000	30.9	NR	NR	88.6	
					2000	48.2	NR	NR	64.9	
					2000	80.5	NR	NR	25	
PEI/TMC/Noria-PG&DSD interlayer/PSF	Negative	295	3.69	10	2000	20	NR	NR	24.61	
					4000	20	NR	NR	10	
					6000	20	NR	NR	4.3	

					2000	30.9	NR	NR	24.61	
					2000	48.2	NR	NR	15.3	
					2000	80.5	NR	NR	4.1	
PEI/TMC/PSF	Negative	388	NR	6	2000	20	NR	NR	5.87	18
Methanol-treated/PEI/TMC/PSF	Negative	579	NR	6	2000	20	NR	NR	5.77	
PEI/TMC/DETA-1/PSF	Negative	534	NR	6	2000	20	NR	NR	9.63	
	Negative	443	NR	6	2000	20	17.25	2.875	11.38	
PEI/TMC/PSF	Negative	263	4.43	10	2000	20	NR	NR	7.1	19
PEI/TMC/CA&PEI interlayer/PSF	Negative	226	4.01	10	2000	20	NR	NR	50.7	
PEI/TMC/HQ&PEI interlayer/PSF		193	3.82	10	2000	20	NR	NR	66.4	
PEI/TMC/PG&PEI interlayer/PSF		204	3.9	10	2000	20	NR	NR	49.4	
PEI&PIP/TMC/PES	Janus	462	0.46	2	2000	30	21.2	10.6	18.26	20
PEI/TMC/PES	Positive	NR	NR	8	2000	30	24	3	7.08	21
PEI/TMC/CNC Interlayer/PES	Janus	NR	NR	8	2000	30	26.96	3.37	8.33	
		NR	NR		2000	60	19.52	2.44	5.79	
		NR	NR		2000	30	33.36	4.17	12.14	
		NR	NR		2000	60	27.2	3.4	5.84	
EDA/TMC/PES LLIP	Negative	220.25	4.4	6	2100	28.68	NR	NR	NR	22
EDA/TMC/PES GLIP-2	Positive	1784.95	11.5	6			NR	NR	NR	
EDA/TMC/PES GLIP-5	Positive	858.96	7.7	6			NR	NR	NR	
EDA/TMC/PES GLIP-10	Positive	640.63	6.4	6			NR	NR	NR	
EDA/TMC/PES GLIP-20	Positive	514.23	5.7	6			NR	NR	NR	
PIP/TMC/PES-SPSF	Negative	392	9.9	5	2000	20	46.4	9.28	4.7	23
PIP/TMC/PES-SPSF-grafted-AS	Negative	382	9.8	5	2000	20	47.2	9.44	24.7	
PIP/TMC/PES-SPSF-grated-AS-Fe	Negative	376	9.7	5	2000	20	48	9.6	81.5	

PIP/TMC/PSF	Negative	327	1.81	NR	NR	NR	NR	NR	NR	24
PIP/TMC/UIO-66-NH_2-doped-PSF-0.3wt%	Negative	300	1.66	10	5500	15.3	881.7	88.17	26.8	
	Negative				10500	30.6	423.6	42.36	26.8	
PIP/TMC/UIO-66-NH_2-doped-PSF-0.6wt%	Negative	268	1.71	10	5500	15.3	897.1	89.71	77.2	
	Negative				10500	30.6	447.1	44.71	78.6	
DAPP/TMC/PAN-HF	Positive	850	NR	3	2000	20	NR	NR	27	25
NF90	Negative	850	NR	3	2000	20	NR	NR	2.1	
PIP/TMC/PES	Negative	361	4.55	10	2000	21.4	76.11	7.61	1.6	26
PIP/TMC-AB_2/PES	Negative	291	3.95	10	2000	21.4	127.3	12.73	35.67	
PIP/TMC/PAN	Negative	800	4.8	6	2100	20	32.2	5.37	1.92	27
[MimAP][Tf2N]-PIP/TMC/PAN	Positive	380	3.2	6	2100	20	28.3	4.72	8.12	
	Positive				4528.1	10	34.08	5.68	9.57	
	Positive				16280	40	23.19	3.87	6.19	
BPEI&PEI/TMC/PEI	Positive	292	2.1	10	10012.3	24	6.3	0.63	3.8	28
	Positive	292	2.1	10	10012.3	24	5.8	0.58	6.5	
EDTA-grafted-BPEI/TMC/PEI	Positive	278	2	10	10012.3	24	6	0.6	9.2	
PEI/TMC/PES	Positive	468	5.58	3	2000	20	15.06	5.02	20	29
GQD-NH2-PEI/TMC/PES	Positive	639	6.38	3	2000	20	42.03	14.01	27.86	
PEI/TMC/PES	Positive	468	NR	3	2000	20	15.06	5.02	15.05	30
MWCNTs-COOK&PEI/TMC/PES	Positive	NR	NR	3	2000	20	24.57	8.19	19.8	
	Positive	NR	NR	3	2000	20	33.51	11.17	32.42	
	Positive	443	NR	3	2000	20	36.69	12.23	57.66	
	Positive	NR	NR	3	2000	20	37.68	12.56	17.47	
	Positive	551	NR	3	2000	20	37.23	12.41	12.51	
PIP-CNTs/-PEI/TMC/PES	Positive	285	4.7	4	2000	21.4	34	8.5	16.46	31

	Positive				130100	21.34	0.5	0.13	7.11	
BHC-CN&BAPP/TMC/PES	Positive	484	NR	4	2000	73	NR	NR	23.9	32
15C5&PEI/TMC/mPSf	Negative	NR	NR	5	2000	5	NR	NR	11.3	33
		NR	NR	5	2000	10	NR	NR	12.5	
		NR	NR	5	2000	20	40	8	11.9	
		NR	NR	5	2000	50	40	8	11.8	
PEI/TMC/PSF	Positive	726.7	0.68	NR	NR	NR	NR	NR	NR	34
DA18C6&PEI/TMC/PSF	Positive	407	0.64	6	2000	20	10.4	NR	10.4	
DA18C6&PEI/TMC/PSF					2000	50	10.4	NR	11.2	
PEI/TBB/mPSf	Positive	369	6.5	3	2000	20	12.6	4.2	16.7	35
PEI&γ-CDs/TMC/PES	Positive	NR	NR	3	2000	30	13.8	4.6	10.8	36
PEI/TMC-QTHIM/PSF	Positive	NR	NR	6	2000	50	170	NR	4.59	37
NaOH-PEI/TMC/PES	Positive	547	3.6	6	2000	20	92.4	15.4	5.3	38
					2000	50	91.8	15.3	5.5	
					2000	100	91.8	15.3	5.7	
SOH-PEI/TMC/PES	Positive	576	4	6	2000	20	145.8	24.3	9	
					2000	50	144	24	9.8	
					2000	100	142.8	23.8	11	
QBPD-PEI/TMC/PSF	Positive	NR	NR	6	2000	50	96.6	16.1	5.2	39
HMTAB-PEI/TMC/PSF	Positive	646	4.6	6	2000	50	97.9	16.32	10.1	40
QEDTP-PEI/TMC/PSF	Positive	508	5.3	6	2000	120	112.9	18.82	15.6	41
GRT&PEI/TMC/PSF	Negative	275	NR	6	2000	5	93.9	15.65	2.3	42
		308	NR	6	2000	20	100.8	16.8	22.7	
		333	NR	6	2000	100	83.7	13.95	34.8	
8NH2-POSS&PEI/TMC/PES	Positive	382	NR	4	2000	50	38.48	9.62	11.6	43

DHTAB/PEI/TMC/PES	Positive	NR	NR	4	2000	42	NR	NR	58.6	44
		NR	NR	4	2000	5	NR	NR	67.8	
		NR	NR	4	2000	10	NR	NR	60	
		NR	NR	4	2000	61	NR	NR	60.1	
		NR	NR	4	2000	40	NR	NR	59.6	
PEI/GA/PAN	Positive	NR	NR	5	1000	10	17	3.4	10.97	45
		NR	NR	5	1000	30	12.45	2.49	7.06	
		NR	NR	5	1000	50	7.4	1.48	5.03	
PEI-LDH/GA/PAN	Positive	NR	NR	5	1000	10	16.5	3.3	18.7	
		NR	NR	5	1000	30	11.95	2.39	9.9	
		NR	NR	5	1000	50	7.4	1.48	5.1	
PEI/TMC/PES	Negative	NR	NR	8	2000	20	43.18	5.4	2.85	46
		NR	NR	8	2000	20	41.17	5.15	4.51	
		NR	NR	8	2000	20	66.95	8.37	2.88	
		NR	NR	8	2000	20	72.64	9.08	2.41	
		NR	NR	8	2000	20	74.31	9.29	2.3	
		NR	NR	8	2000	20	78.33	9.79	1.45	
		NR	NR	8	2000	20	74.67	9.33	5	
		NR	NR	8	2000	20	54.33	6.79	7.5	
		NR	NR	8	2000	20	45	5.63	7.74	
		NR	NR	8	2000	20	40	5	17.14	
		NR	NR	8	2000	20	28.67	3.58	8	
		NR	NR	8	2000	20	29.33	3.67	6.15	
		NR	NR	8	2000	20	80.08	10.01	3.6	
		NR	NR	8	2000	20	75.36	9.42	4.46	

		NR	NR	8	2000	20	40.24	5.03	17.15	
		NR	NR	8	2000	20	42.16	5.27	7.19	
		NR	NR	8	2000	20	50.48	6.31	4.65	
		NR	NR	8	2000	20	81.28	10.16	2.65	
		NR	NR	8	2000	20	42.59	5.32	2.34	
		NR	NR	8	2000	20	41.73	5.22	2.64	
		NR	NR	8	2000	20	41.51	5.19	3.47	
		NR	NR	8	2000	20	41.08	5.14	4.04	
		NR	NR	8	2000	20	40.65	5.08	7.93	
		NR	NR	8	2000	20	28.97	3.62	7.93	
		NR	NR	8	2000	20	30.92	3.86	6.34	
Cu-MPD	Positive	NR	NR	5	2000	23.5	80.8	16.16	8	[47]
Polyester	Positive	389	2.6	6	2000	50	100	16.67	28	[48]
(PSS/PAH)2.5	Negative	198	NR	4	2000	20	40	10	279	[49]
			NR	4	2000	40	32	8	370.3	
			NR	4	2000	60	31.6	7.9	381.6	
			NR	4	4000	20	NR	NR	250.2	
			NR	4	5000	20	NR	NR	249.2	
			NR	4	6000	20	NR	NR	153.2	
(PSS/PAH)2.5-X	Negative	171	NR	4	2000	20	40	10	262.3	
			NR	4	2000	40	32	8	255.2	
			NR	4	2000	60	31.6	7.9	330.2	
			NR	4	4000	20	NR	NR	196.2	
			NR	4	5000	20	NR	NR	176.7	
			NR	4	6000	20	NR	NR	126	

(PSS/PAH)2.5-H	Negative	188	NR	4	2000	20	43.6	10.9	81.1	50
(PSS/PAH)2.5-H		188	NR	4	2000	40	42.4	10.6	78.5	
(PSS/PAH)2.5-H		188	NR	4	2000	60	40	10	87.2	
AMN/PEI-TMC	Positive	440	4.9	6	2000	5	45.48	11.37	30.18	51
		440	4.9	6	2000	10	44.56	11.14	28.73	
		440	4.9	6	2000	20	44.56	11.14	26.57	
		440	4.9	6	2000	50	44.08	11.02	25.12	

*NR stands for not reported.

S4. Typical Salt-lake Brines Composition.

Table S2. The composition of typical salt-lake brines worldwide.[52,53]

Salt Lake, Location	Composition ($g \cdot L^{-1}$)							Mg^{2+}/Li^{+} ratio	Total salt concentration ($g \cdot L^{-1}$)
	Li^+	Na^+	K^+	Mg^{2+}	Ca^{2+}	Cl^-	SO_4^{2-}		
Salar De Atacama, Chile	1.19	69.01	17.89	7.31	NR	143.72	12.06	6.15	251.18
Salar De Maricunga, Chile	0.90	71.00	6.90	6.10	11.24	159.10	0.60	6.78	255.84
Great Salt Lake, Bolivia	0.018	37.00	2.600	5.00	0.26	70.00	9.40	277.78	124.35
Salar De Uyuni, Bolivia	0.32	70.60	11.70	6.50	0.31	50.00	NR*	20.25	140.14
Hombre Muerto, Argentina	0.60	97.90	13.70	0.90	0.50	158.00	8.50	1.50	280.10
Cauchari-Olaroz, Argentina	0.50	95.5	4.70	1.31	0.30	148.6	16.20	2.62	267.11
Qaidam Lake, China	0.31	56.30	4.40	20.20	NR	134.20	31.40	65.16	249.51
Qarhan Lake, China	0.01	59.50	10.90	23.90	0.85	168	5.30	2390.00	268.46
East Taijinar Lake, China	0.85	51.3	14.7	29.9	0.20	183.81	NR	35.17	280.76
West Taijinar Lake, China	0.21	84.3	7.00	13.10	0.16	152.90	29.40	62.38	287.07
Yiliping Lake, China	0.15	66.3	8.20	14.50	0.60	160.70	5.40	96.67	255.85
Zabuye Salt Lake, China	0.80	98.10	20.5	0.02	NR	117.80	46.70	0.025	283.92
Jiezechaka Lake, China	1.17	40.90	2.42	0.33	NR	61.90	2.92	0.28	109.64

Longmu Co Lake, China	0.79	29.20	3.38	11.23	0.06	82.40	NR	14.22	127.06
Silver Park, United States	0.40	62.00	8.00	0.40	0.50	100.6	7.10	1.00	179.00
Salton See, United States	0.22	53.70	NR	17.10	26.3	152.00	0.12	77.73	249.44
Clayton Valley, United States	0.163	46.90	4.00	0.19	0.45	72.60	3.40	1.166	127.75

*NR stands for not reported.

References

(1) Friedman, J. H. Greedy Function Approximation: A Gradient Boosting Machine. *Ann. Statist.* **2001**, *29* (5).

(2) Chen, T.; Guestrin, C. XGBoost: A Scalable Tree Boosting System. In *Proceedings of the 22nd ACM SIGKDD International Conference on Knowledge Discovery and Data Mining*; ACM: San Francisco California USA, 2016; pp 785–794.

(3) Nielsen, D. Tree Boosting With XGBoost.

(4) Bischl, B.; Richter, J.; Bossek, J.; Horn, D.; Thomas, J.; Lang, M. mlrMBO: A Modular Framework for Model-Based Optimization of Expensive Black-Box Functions. arXiv December 3, 2018.

(5) Lundberg, S.; Lee, S.-I. A Unified Approach to Interpreting Model Predictions. arXiv November 24, 2017.

(6) Liu, Y.; Just, A. SHAPforxgboost: SHAP Plots for "XGBoost," 2019, 0.1.3.

(7) Yang, Z.; Long, L.; Wu, C.; Tang, C. Y. High Permeance or High Selectivity? Optimization of System-Scale Nanofiltration Performance Constrained by the Upper Bound. *ACS EST Eng.* **2022**, *2* (3), 377–390.

(8) Zhang, Y.; Fan, Y.; Zhou, G.; Cao, Y.; Wang, J.; Jiang, X.; Zhang, N.; Yin, S. Based on High Cross-Linked Structure Design to Fabricate PEI-Based Nanofiltration Membranes for Mg^{2+}/Li^{+} Separation. *J. Membr. Sci.* **2024**, *693*, 122351.

(9) Zhao, S.; Cui, W.; Shen, Q.; Yao, Z.; Fang, C.; Zhang, L.; Zhu, L. Porous Organic Polymer Interlayers Modulated Nanofiltration Membranes for Ultra-Permselective Li^{+}/Mg^{2+} Separation. *J. Membr. Sci.* **2024**, *690*, 122207.

(10) Guo, B.-B.; Liu, C.; Zhu, C.-Y.; Xin, J.-H.; Zhang, C.; Yang, H.-C.; Xu, Z.-K. Double Charge Flips of Polyamide Membrane by Ionic Liquid-Decoupled Bulk and Interfacial Diffusion for on-Demand Nanofiltration. *Nat. Commun.* **2024**, *15* (1), 2282.

(11) Yuan, B.; Zhang, Y.; Qi, P.; Yang, D.; Hu, P.; Zhao, S.; Zhang, K.; Zhang, X.; You, M.; Cui, J.; Jiang, J.; Lou, X.; Niu, Q. J. Self-Assembled Dendrimer Polyamide Nanofilms with Enhanced Effective Pore Area for Ion Separation. *Nat. Commun.* **2024**, *15* (1), 471.

(12) Li, T.; Zhang, X.; Zhang, Y.; Wang, J.; Wang, Z.; Zhao, S. Nanofiltration Membrane Comprising Structural Regulator Cyclen for Efficient Li^{+}/Mg^{2+} Separation. *Desalination* **2023**, *556*, 116575.

(13) Xu, P.; Gonzales, R. R.; Hong, J.; Guan, K.; Chiao, Y.-H.; Mai, Z.; Li, Z.; Rajabzadeh, S.; Matsuyama, H. Fabrication of Highly Positively Charged Nanofiltration Membranes by Novel Interfacial Polymerization: Accelerating Mg^{2+} Removal and Li^{+} Enrichment. *J. Membr. Sci.* **2023**, *668*, 121251.

(14) Setiawan, O.; Huang, Y.-H.; Abdi, Z. G.; Hung, W.-S.; Chung, T.-S. pH-Tunable and pH-Responsive Polybenzimidazole (PBI) Nanofiltration Membranes for Li^{+}/Mg^{2+} Separation. *J. Membr. Sci.* **2023**, *668*, 121269.

(15) Wu, H.; Zhao, H.; Lin, Y.; Liu, X.; Wang, L.; Yao, H.; Tang, Y.; Yu, L.; Wang, H.; Wang, X. Positively-Charged PEI/TMC Nanofiltration Membrane Prepared by Adding a Diamino-Silane Coupling Agent for Li^{+}/Mg^{2+} Separation. *J. Membr. Sci.* **2023**, *672*, 121468.

(16) Fang, S.; Guan, K.; Zhou, S.; Song, Q.; Shi, Y.; Fu, W.; Li, Z.; Xu, P.; Hu, M.; Mai, Z.; Zhang, P.; Matsuyama, H. Ternary-Coordination-Regulated Polyamide Nanofiltration Membranes for Li^{+}/Mg^{2+} Separation. *Desalination* **2024**, *581*, 117577.

(17) Chen, K.; Li, F.; Wei, T.; Zhou, H.; Zhang, T.; Zhao, S.; Xie, T.; Sun, H.; Li, P.; Niu, Q. J. An Interlayer-Based Positive Charge Compensation Strategy for the Preparation of Highly Selective Mg^{2+}/Li^{+} Separation Nanofiltration Membranes. *J. Membr. Sci.* **2023**, *684*, 121882.

(18) Li, H.; Li, Y.; Li, M.; Jin, Y.; Kang, G.; Cao, Y. Improving Mg^{2+}/Li^{+} Separation Performance of Polyamide Nanofiltration Membrane by Swelling-Embedding-Shrinking Strategy. *J. Membr. Sci.* **2023**, *669*, 121321.

(19) Chen, K.; Zhao, S.; Lan, H.; Xie, T.; Wang, H.; Chen, Y.; Li, P.; Sun, H.; Niu, Q. J.; Yang, C. Dual-Electric Layer Nanofiltration Membranes Based on Polyphenol/PEI Interlayer for Highly Efficient Mg^{2+}/Li^{+} Separation. *J. Membr. Sci.* **2022**, *660*, 120860.

(20) Guo, C.; Qian, Y.; Liu, P.; Zhang, Q.; Zeng, X.; Xu, Z.; Zhang, S.; Li, N.; Qian, X.; Yu, F. One-Step Construction of the Positively/Negatively Charged Ultrathin Janus Nanofiltration Membrane for the Separation of Li^{+} and Mg^{2+}. *ACS Appl. Mater. Interfaces* **2023**, *15* (3), 4814–4825.

(21) Guo, C.; Li, N.; Qian, X.; Shi, J.; Jing, M.; Teng, K.; Xu, Z. Ultra-Thin Double Janus Nanofiltration Membrane for Separation of Li^{+} and Mg^{2+}: “Drag” Effect from Carboxyl-Containing Negative Interlayer. *Sep. Purif. Technol.* **2020**, *230*, 115567.

(22) Wu, M.-B.; Ye, H.; Zhu, Z.-Y.; Chen, G.-T.; Ma, L.-L.; Liu, S.-C.; Liu, L.; Yao, J.; Xu, Z.-K. Positively-Charged Nanofiltration Membranes Constructed via Gas/Liquid Interfacial Polymerization for Mg^{2+}/Li^{+} Separation. *J. Membr. Sci.* **2022**, *644*, 119942.

(23) Liu, Y.; Li, Q.; Wang, S.; Liang, M.; Ji, Y.; Cui, Z.; Younas, M.; Li, J.; He, B. A Nanofiltration Membrane with Positively and Negatively Charged Groups by Grafted P-Aminosalicylic Acid-Fe(III) Chelation for Li^{+}/Mg^{2+} Efficient Separation. *Sep. Purif. Technol.* **2023**, *308*, 122968.

(24) Yuan, B.; Wang, N.; Zhao, S.; Hu, P.; Jiang, J.; Cui, J.; Zhang, X.; You, M.; Lou, X.; Niu, Q. J. Polyamide Nanofiltration Membrane Fine-Tuned via Mixed Matrix Ultrafiltration Support to Maximize the Sieving Selectivity of Li^{+}/Mg^{2+} and Cl^{-}/SO_4^{2-}. *Desalination* **2022**, *538*, 115929.

(25) Li, X.; Zhang, C.; Zhang, S.; Li, J.; He, B.; Cui, Z. Preparation and Characterization of Positively Charged Polyamide Composite Nanofiltration Hollow Fiber Membrane for Lithium and Magnesium Separation. *Desalination* **2015**, *369*, 26–36.

(26) Hu, P.; Yuan, B.; Niu, Q. J.; Chen, K.; Xu, Z.; Tian, B.; Zhang, X. Modification of Polyamide Nanofiltration Membrane with Ultra-High Multivalent Cations Rejections and Mono-/Divalent Cation Selectivity. *Desalination* **2022**, *527*, 115553.

(27) Wu, H.; Lin, Y.; Feng, W.; Liu, T.; Wang, L.; Yao, H.; Wang, X. A Novel Nanofiltration Membrane with [MimAP][Tf2N] Ionic Liquid for Utilization of Lithium from Brines with High Mg^{2+}/Li^{+} Ratio. *J. Membr. Sci.* **2020**, *603*, 117997.

(28) Li, W.; Shi, C.; Zhou, A.; He, X.; Sun, Y.; Zhang, J. A Positively Charged Composite Nanofiltration Membrane Modified by EDTA for $LiCl/MgCl_2$ Separation. *Sep. Purif. Technol.* **2017**, *186*, 233–242.

(29) Xu, P.; Hong, J.; Xu, Z.; Xia, H.; Ni, Q.-Q. Novel Aminated Graphene Quantum Dots (GQDs-NH2)-Engineered Nanofiltration Membrane with High Mg^{2+}/Li^{+} Separation Efficiency. *Sep. Purif. Technol.* **2021**, *258*, 118042.

(30) Xu, P.; Hong, J.; Xu, Z.; Xia, H.; Ni, Q.-Q. MWCNTs-COOK-Assisted High Positively Charged Composite Membrane: Accelerating Li^{+} Enrichment and Mg^{2+} Removal. *Compos. B Eng.* **2021**, *212*, 108686.

(31) Zhang, H.-Z.; Xu, Z.-L.; Ding, H.; Tang, Y.-J. Positively Charged Capillary Nanofiltration Membrane with High Rejection for Mg^{2+} and Ca^{2+} and Good Separation for Mg^{2+} and Li^{+}. *Desalination* **2017**, *420*, 158–166.

(32) Bi, Q.; Zhang, C.; Liu, J.; Liu, X.; Xu, S. Positively Charged Zwitterion-Carbon Nitride Functionalized Nanofiltration Membranes with Excellent Separation Performance of Mg^{2+}/Li^{+} and Good Antifouling Properties. *Sep. Purif. Technol.* **2021**, *257*, 117959.

(33) Dong, Y.; Liu, Y.; Li, H.; Zhu, Q.; Luo, M.; Zhang, H.; Ye, B.; Yang, Z.; Xu, T. Crown Ether-Based Tröger's Base Membranes for Efficient Li^{+}/Mg^{2+} Separation. *J. Membr. Sci.* **2023**, *665*, 121113.

(34) Zha, Z.; Li, T.; Hussein, I.; Wang, Y.; Zhao, S. Aza-Crown Ether-Coupled Polyamide Nanofiltration Membrane for Efficient Li^{+}/Mg^{2+} Separation. *J. Membr. Sci.* **2024**, *695*, 122484.

(35) Zhao, Z.; Di, N.; Zha, Z.; Wang, J.; Wang, Z.; Zhao, S. Positively Charged Polyamine Nanofiltration Membrane for Precise Ion–Ion Separation. *ACS Appl. Mater. Interfaces* **2023**, *15* (41), 48695–48704.

(36) Zhao, Y.; Li, N.; Shi, J.; Xia, Y.; Zhu, B.; Shao, R.; Min, C.; Xu, Z.; Deng, H. Extra-Thin Composite Nanofiltration Membranes Tuned by γ-Cyclodextrins Containing Amphipathic Cavities for Efficient Separation of Magnesium/Lithium Ions. *Sep. Purif. Technol.* **2022**, *286*, 120419.

(37) Liu, X.; Feng, Y.; Ni, Y.; Peng, H.; Li, S.; Zhao, Q. High-Permeance Mg^{2+}/Li^{+} Separation Nanofiltration Membranes Intensified by Quadruple Imidazolium Salts. *J. Membr. Sci.* **2023**, *667*, 121178.

(38) Peng, H.; Hu, Y.; Li, S.; Rao, J.; Zhao, Q. Sulfonium-Polyamide Membranes for High Flux Mg^{2+}/Li^{+} Separation. *J. Membr. Sci.* **2023**, *674*, 121515.

(39) Feng, Y.; Peng, H.; Zhao, Q. Fabrication of High Performance Mg^{2+}/Li^{+} Nanofiltration Membranes by Surface Grafting of Quaternized Bipyridine. *Sep. Purif. Technol.* **2022**, *280*, 119848.

(40) Luo, H.; Peng, H.; Zhao, Q. High Flux Mg^{2+}/Li^{+} Nanofiltration Membranes Prepared by Surface Modification of Polyethylenimine Thin Film Composite Membranes. *Appl. Surf. Sci.* **2022**, *579*, 152161

(41) Xu, Y.; Peng, H.; Luo, H.; Zhang, Q.; Liu, Z.; Zhao, Q. High Performance Mg^{2+}/Li^{+} Separation Membranes Modified by a Bis-Quaternary Ammonium Salt. *Desalination* **2022**, *526*, 115519.

(42) Yang, N.; Li, K.; Hao, Y.; Zhang, L.; Sun, Y.; Zhang, L.; Jiang, B. Advanced Mg^{2+}/Li^{+} Separation Nanofiltration Membranes Fabricated with Girard's Reagent T Based on Functional End-Capping Strategy. *J. Membr. Sci.* **2024**, *695*, 122483.

(43) Zhang, S.; Jiang, Y.; Yue, X.; Zhang, R.; Li, R.; Gu, T.; Wu, T.; Zhao, J.; Zhang, S.; Jiang, Z. Bifunctional Polyhedral Oligomeric Silsesquioxane Engineered Polyamide Membrane for Efficient Li^+/Mg^{2+} Separation. *Sep. Purif. Technol.* **2023**, *327*, 124875.

(44) Gu, T.; Zhang, R.; Zhang, S.; Shi, B.; Zhao, J.; Wang, Z.; Long, M.; Wang, G.; Qiu, T.; Jiang, Z. Quaternary Ammonium Engineered Polyamide Membrane with High Positive Charge Density for Efficient Li^+/Mg^{2+} separation. *J. Membr. Sci.* **2022**, *659*, 120802.

(45) Ni, H.; Wang, N.; Yang, Y.; Shen, M.; An, Q.-F. Positively-Charged Nanofiltration Membrane Constructed by Polyethyleneimine/Layered Double Hydroxide for Mg^{2+}/Li^+ Separation. *Desalination* **2023**, *548*, 116256.

(46) Xu, P.; Wang, W.; Qian, X.; Wang, H.; Guo, C.; Li, N.; Xu, Z.; Teng, K.; Wang, Z. Positive Charged PEI-TMC Composite Nanofiltration Membrane for Separation of Li^+ and Mg^{2+} from Brine with High Mg^{2+}/Li^+ Ratio. *Desalination* **2019**, *449*, 57–68.

(47) Wang, L.; Rehman, D.; Sun, P.-F.; Deshmukh, A.; Zhang, L.; Han, Q.; Yang, Z.; Wang, Z.; Park, H.-D.; Lienhard, J. H.; Tang, C. Y. Novel Positively Charged Metal-Coordinated Nanofiltration Membrane for Lithium Recovery. *ACS Appl. Mater. Interfaces* **2021**, *13* (14), 16906–16915.

(48) Li, J.; Peng, H.; Liu, K.; Zhao, Q. Polyester Nanofiltration Membranes for Efficient Cations Separation. *Adv. Mater.* **2024**, *36* (9), 2309406.

(49) He, R.; Xu, S.; Wang, R.; Bai, B.; Lin, S.; He, T. Polyelectrolyte-Based Nanofiltration Membranes with Exceptional Performance in Mg^{2+}/Li^+ Separation in a Wide Range of Solution Conditions. *J. Membr. Sci.* **2022**, *663*, 121027.

(50) He, R.; Dong, C.; Xu, S.; Liu, C.; Zhao, S.; He, T. Unprecedented Mg^{2+}/Li^+ Separation Using Layer-by-Layer Based Nanofiltration Hollow Fiber Membranes. *Desalination* **2022**, *525*, 115492.

(51) Li, J.; Fang, L.; Xu, D.; Zhang, X.; Jiang, L.; Zhu, Q.; Chen, Q.; Jin, P.; Volodine, A.; Dewil, R.; Gui, X.; Gao, Q.; Van Der Bruggen, B. Intercalation of Small Molecules in the Selective Layer of Polyamide Nanofiltration Membranes Facilitates the Separation of Mg^{2+}/Li^+. *J. Chem. Eng.* **2024**, *487*, 150659.

(52) Vera, M. L.; Torres, W. R.; Galli, C. I.; Chagnes, A.; Flexer, V. Environmental Impact of Direct Lithium Extraction from Brines. *Nat. Rev. Earth Environ.* **2023**, *4* (3), 149–165.

(53) Xu, S.; Song, J.; Bi, Q.; Chen, Q.; Zhang, W.-M.; Qian, Z.; Zhang, L.; Xu, S.; Tang, N.; He, T. Extraction of Lithium from Chinese Salt-Lake Brines by Membranes: Design and Practice. *J. Membr. Sci.* **2021**, *635*, 119441.
